# Systematic Review and Meta-Analysis of RCTs on Efficacy of Conventional vs. Emerging Treatments for Amblyopia

**DOI:** 10.3390/life16020222

**Published:** 2026-01-28

**Authors:** Clara Martinez-Perez, Ana Paula Oliveira

**Affiliations:** 1 Applied Physics Department (Optometry Area), Facultade de Óptica e Optometría, Universidade de Santiago de Compostela, 15705 Santiago de Compostela, Spain; 2 Instituto Superior de Educação e Ciências de Lisboa (ISEC Lisboa), Alameda das Linhas de Torres, 1750-142 Lisboa, Portugal; ana.oliveira@iseclisboa.pt; 3 Centro de Investigação, Desenvolvimento e Inovação em Turismo (CiTUR)—Polo Estoril, Avenida Condes de Barcelona, 2769-510 Estoril, Portugal

**Keywords:** amblyopia, occlusion therapy, atropine penalization, dichoptic therapy, virtual reality, digital therapy, pharmacological adjuvants

## Abstract

Amblyopia affects 1–4% of the population and remains a leading cause of unilateral visual impairment, with adherence and residual deficits limiting outcomes of standard therapies. This systematic review and meta-analysis compared the effectiveness of conventional and emerging amblyopia treatments in children, adolescents, and adults with anisometropic, strabismic, or mixed amblyopia. Following PRISMA guidelines and PROSPERO registration (CRD420251123552), PubMed, Web of Science, and Scopus were searched up to 5 August 2025 for randomized controlled trials. Sixty-six trials (sample sizes 7–404) were included, with thirty-six contributing to the meta-analysis. Primary outcomes were best-corrected visual acuity (logMAR) and stereopsis. Risk of bias was assessed using the Cochrane tool, and certainty of evidence was assessed using GRADE. Atropine penalization and occlusion demonstrated equivalent effects on visual acuity (mean difference 0.04 logMAR; 95% CI −0.04 to 0.12; moderate-certainty evidence). Digital, dichoptic, binocular, and virtual reality therapies showed a statistically significant but small improvement over patching (mean difference 0.02 logMAR; 95% CI 0.00–0.04; low-certainty evidence). Pharmacological adjuvants combined with patching yielded slightly larger gains (mean difference 0.08 logMAR; 95% CI 0.03–0.13; low-to-moderate certainty). No consistent benefit was observed for stereopsis outcomes. Overall, the certainty of evidence ranged from low to moderate, and most pooled effects were below commonly accepted thresholds for clinically meaningful visual acuity improvement (≈0.1 logMAR, one line). Atropine and occlusion remain equivalent first-line treatments, while adjunctive and multimodal approaches may offer limited additional benefit in selected patients when adherence, tolerability, and engagement are prioritized.

## 1. Introduction

Amblyopia is one of the most common causes of unilateral vision impairment in children, with an estimated prevalence of 1–4% worldwide [1,2,3,4]. It is characterized by reduced visual efficiency in one eye without the presence of an organic cause or to a degree disproportionate to any existing organic defect [5]. The principal etiologies include significant refractive error (either asymmetric, causing unilateral amblyopia, or high bilateral error), strabismus, and early visual deprivation, most commonly congenital [6]. Amblyopia is typically diagnosed around the age of 3–5 years, and initial treatment consists of refractive correction, with occlusion therapy recommended if similar visual acuity is not achieved after three months [7,8].

Traditionally, the mainstays of amblyopia treatment have been occlusion of the non-amblyopic eye with an adhesive eye patch and penalization using atropine eye drops [5,9,10]. Both approaches aim to force use of the amblyopic eye, promoting functional recovery [5]. Although both patching and atropine are effective for improving visual acuity, a considerable proportion of children are left with residual decreased visual acuity or fail to respond to treatment [9,10,11,12,13].

Adherence to amblyopia therapy has emerged as a crucial determinant of treatment outcomes [13,14,15]. Objective measurement of adherence using occlusion dose monitors (ODMs) has demonstrated that the actual occlusion dose is often lower than prescribed, with reported adherence rates ranging from 40% to 57% [16,17,18]. A positive linear relationship between total occlusion dose and visual outcome has been observed, particularly within the first 400 h of treatment [17]. Factors such as discomfort, social stigma, skin irritation, cosmetic concerns, and the continuous occlusion of the non-amblyopic eye significantly contribute to poor adherence [19,20]. Sociodemographic variables, including parental fluency in the national language, educational level, and amblyopia severity, have also been identified as significant predictors of adherence [16,19]. Notably, younger children typically exhibit higher adherence than older children [16].

The Pediatric Eye Disease Investigator Group (PEDIG) has played a pivotal role in advancing knowledge of amblyopia treatment by conducting prospective clinical trials addressing questions regarding the duration and intensity of occlusion therapy [21,22,23]. Early beliefs favored full-time patching, but more recent studies have examined the efficacy of part-time occlusion, with daily prescriptions ranging from 1 to 24 h [21,24]. Evidence suggests that augmenting optical correction with two to six hours of daily patching can double response rates in children aged 7–12 years [23]. However, the impact of age on treatment efficacy remains debated, with studies reporting conflicting results and some demonstrating substantial improvement even beyond 12 years of age [25,26,27]. Atropine remains a common cycloplegic agent, particularly in children with dark irides, as it is believed to more fully reveal latent hyperopia compared to cyclopentolate, despite the need for multiple administrations and challenges with parental compliance [28,29]. Non-compliance with the atropine protocol, sometimes exceeding 48%, can compromise refractive assessment accuracy and treatment outcomes [30]. Additionally, factors such as ethnicity, skin color, and even crying during instillation have been considered influential in cycloplegic efficacy and patient experience [31,32,33]. The past decade has seen the emergence of dichoptic, binocular, and digital therapies, including video games and virtual reality (VR), as alternatives or supplements to standard patching [11,34,35,36,37,38,39,40]. Dichoptic treatments target the underlying binocular dysfunction and interocular suppression characteristic of amblyopia [37,41,42]. Contrast-rebalanced binocular gameplay, in which the fellow eye’s contrast is reduced, has been shown to yield significant improvements in visual acuity, sometimes exceeding those achieved with patching [34,35,37]. The efficiency of digital and dichoptic training appears substantially higher, with 10–20 h of gaming therapy reported to yield equivalent benefits to over 100 h of patching in young amblyopia patients [9,36,37,42].

Nevertheless, barriers to long-term compliance and limited improvement in stereovision remain significant challenges for digital and dichoptic approaches [11,34,35,37,43]. Recent studies suggest that VR-based interventions, which offer immersive and interactive training with 3D cues, may further enhance both adherence and efficacy, particularly regarding stereovision recovery and selective attention deficits [44,45,46,47]. While other systematic reviews and meta-analyses have explored the effectiveness of amblyopia treatments, many have focused on traditional options. For example, Li et al. [48] and Brin et al. [49] conducted network meta-analyses comparing patching, atropine, and binocular therapies but excluded recent digital approaches or pharmacological adjuvants. Other reviews addressed single modalities, such as levodopa, perceptual learning, and video games [50,51] or VR [52], often targeting narrow populations or lacking comparative synthesis. Unlike previous reviews, this meta-analysis integrates a broader and more up-to-date range of interventions, including emerging digital therapies, VR-based programs, and pharmacological adjuvants, using standardized outcomes across visual acuity, stereopsis, and adherence. This comprehensive and comparative approach provides new insights to guide personalized and evidence-based amblyopia management in both clinical and research settings.

## 2. Materials and Methods

### 2.1. Research Question and PICOS Framework

**Figure 1:**
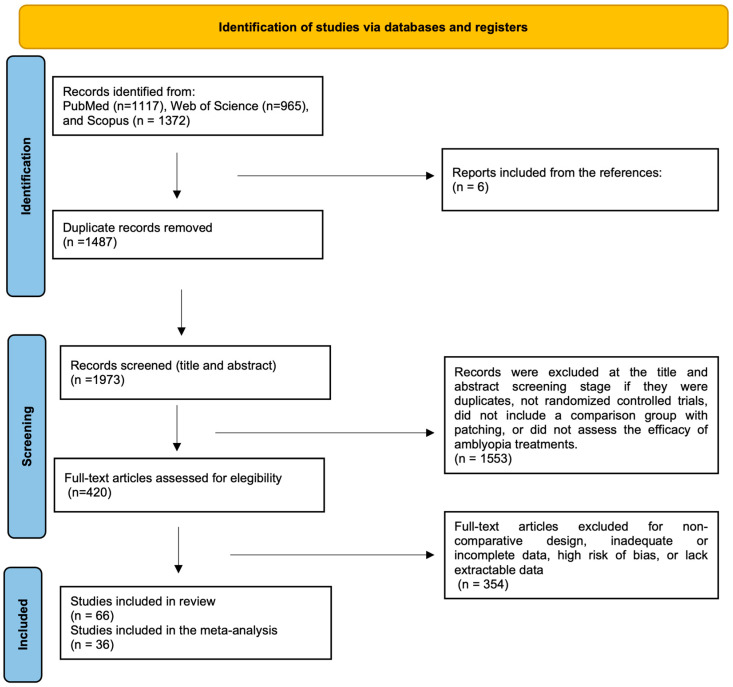
PRISMA flow diagram of study selection.

This systematic review and meta-analysis was registered in PROSPERO (registration number: [CRD420251123552]) and conducted according to PRISMA 2020 [53] guidelines and AMSTAR-2 [54] methodological standards (Figure 1). A completed PRISMA checklist is available as Supplementary Materials (File S1). The final literature search was completed on 5 August 2025.

The research question was formulated using the PICOS framework to ensure methodological rigor and relevance. Specifically, the objective was to evaluate whether children, adolescents, and adults diagnosed with amblyopia (Population) experience greater improvement in visual acuity and other clinically relevant outcomes (Outcomes) when treated with alternative or adjunctive interventions, including dichoptic/binocular/VR/digital therapy, pharmacological adjuvants, perceptual learning, or near activities (Intervention) compared to standard patching, atropine penalization, or optical correction (Comparator). All eligible studies were randomized controlled trials (Study design), and the interventions of interest comprised a range of established and emerging therapies for amblyopia.

Primary outcomes focused on changes in visual acuity and, where available, stereopsis or other functional vision metrics. Secondary outcomes included treatment adherence, adverse effects, and the influence of age, amblyopia subtype, and treatment duration on efficacy. Through this comprehensive approach, our review aimed to synthesize the current evidence on comparative effectiveness of amblyopia treatments, clarify sources of heterogeneity, and provide guidance for clinical decision-making and future research priorities.

### 2.2. Eligibility Criteria

Studies were excluded if they met any of the following criteria: case reports, case series, quasi-experimental designs, or other uncontrolled studies; systematic or narrative reviews; duplicate publications from the same dataset; or those rated as having an overall high risk of bias, defined a priori as high risk in two or more key domains of the Cochrane Risk of Bias tool (random sequence generation, allocation concealment, or incomplete outcome data), or as high risk in a single critical domain judged likely to materially affect the primary outcome. Additional exclusions were applied to studies with non-comparable or incomplete demographic data, unclear or inadequate diagnostic criteria for amblyopia, absence of a randomized control group with patching or other standard interventions, or lack of relevant visual outcomes (e.g., visual acuity, stereopsis) necessary for quantitative synthesis. Studies were also excluded if they did not report sufficient statistical data (such as means and standard deviations or confidence intervals) required for meta-analysis.

### 2.3. Information Sources

A comprehensive and systematic literature search was conducted across three major electronic databases—PubMed, Web of Science, and Scopus—without restrictions on publication date or language. To ensure the inclusion of all relevant evidence, reference lists of relevant reviews and included studies were manually screened. Records identified through this process were added prior to duplicate removal and underwent the same title/abstract screening and full-text eligibility assessment as database-derived records.

### 2.4. Search Methods for Identification of Studies

The search strategy combined controlled vocabulary (Medical Subject Headings [MeSH] in PubMed) and free-text terms related to amblyopia and its major treatment modalities. Searches were conducted in PubMed, Web of Science, and Scopus, with the final search completed on 5 August 2025. No restrictions on publication date or language were applied, and studies published in languages other than English were translated when relevant data were available.

Core search concepts included terms related to amblyopia (“amblyopia”, “lazy eye”) combined with treatment-related terms such as “patching”, “occlusion”, “occlusion therapy”, “atropine”, “Bangerter filter”, “binocular therapy”, “dichoptic training”, “virtual reality”, “video game”, “digital game”, “perceptual learning”, “optical correction”, “spectacle correction”, “refractive correction”, “penalization”, and “Luminopia”. These were paired with comparative keywords including “versus”, “comparison”, “head-to-head”, and “compared”. Database-specific adaptations of the search strategy were applied as appropriate for each platform.

Gray literature, clinical trial registries, and conference abstracts were not systematically searched, as this review focused on randomized controlled trials published in peer-reviewed journals with sufficient methodological detail and extractable outcome data to allow quantitative synthesis. A summary of the electronic search strategies, including the databases searched, key search terms, and the date of the last search, is provided in Table 1. Full database-specific search strings are reported in File S2.

### 2.5. Data Extraction and Data Items

Two authors (A.P.O. and C.M.P.) independently extracted data from all eligible randomized controlled trials. For each included study, key characteristics were recorded, including first author’s name, year of publication, country or region, study design, sample size for each intervention group, mean age of participants, type(s) of amblyopia included, treatments compared, treatment duration, diagnostic criteria for amblyopia, and reported conflicts of interest. Any discrepancies in data extraction or eligibility assessment were resolved through discussion and consensus, with no need for a third reviewer. Study management, including duplicate removal and tracking of eligibility decisions, was conducted using Microsoft Excel.

The primary variables extracted focused on visual acuity and stereopsis outcomes, as well as other clinically relevant measures such as adherence, treatment compliance, and adverse events when reported. Additional data on treatment protocols (e.g., patching regimen, dosage of pharmacological agents, type and duration of digital therapy), inclusion/exclusion criteria, and subgroup characteristics (e.g., age ranges, amblyopia subtype, prior treatments) were also collected to facilitate subgroup and sensitivity analyses, and to assess methodological heterogeneity across studies.

### 2.6. Methodological Quality and Risk of Bias Assessment

The methodological quality and risk of bias of the included randomized controlled trials were independently assessed by two reviewers using the Cochrane Collaboration’s Risk of Bias tool, as implemented in Review Manager (RevMan) software. Seven domains were evaluated: random sequence generation, allocation concealment, blinding of participants and personnel (performance bias), blinding of outcome assessment (detection bias), incomplete outcome data (attrition bias), selective reporting (reporting bias), and other potential sources of bias.

Each domain was judged as low, unclear, or high risk of bias according to predefined criteria. A domain was considered critical when judged likely to materially affect the primary outcome (e.g., inadequate allocation concealment or substantial differential attrition). Disagreements between reviewers were resolved by discussion and consensus, without the involvement of a third reviewer.

Overall, most trials were rated at low risk of bias for random sequence generation and selective reporting. Allocation concealment and blinding of participants and personnel were the domains most frequently rated as unclear or high risk, reflecting the inherent difficulty of masking in behavioral and occlusion-based interventions. Attrition bias was generally low, with incomplete outcome data adequately addressed in the majority of studies.

Randomized controlled trials presenting some domains at high or unclear risk of bias were not automatically excluded. Studies were excluded from quantitative synthesis only when predefined criteria for overall high risk of bias were met (see Section 2.2). Trials with mixed risk profiles were retained, and their influence on pooled estimates was explored through sensitivity analyses.

**Figure 2:**
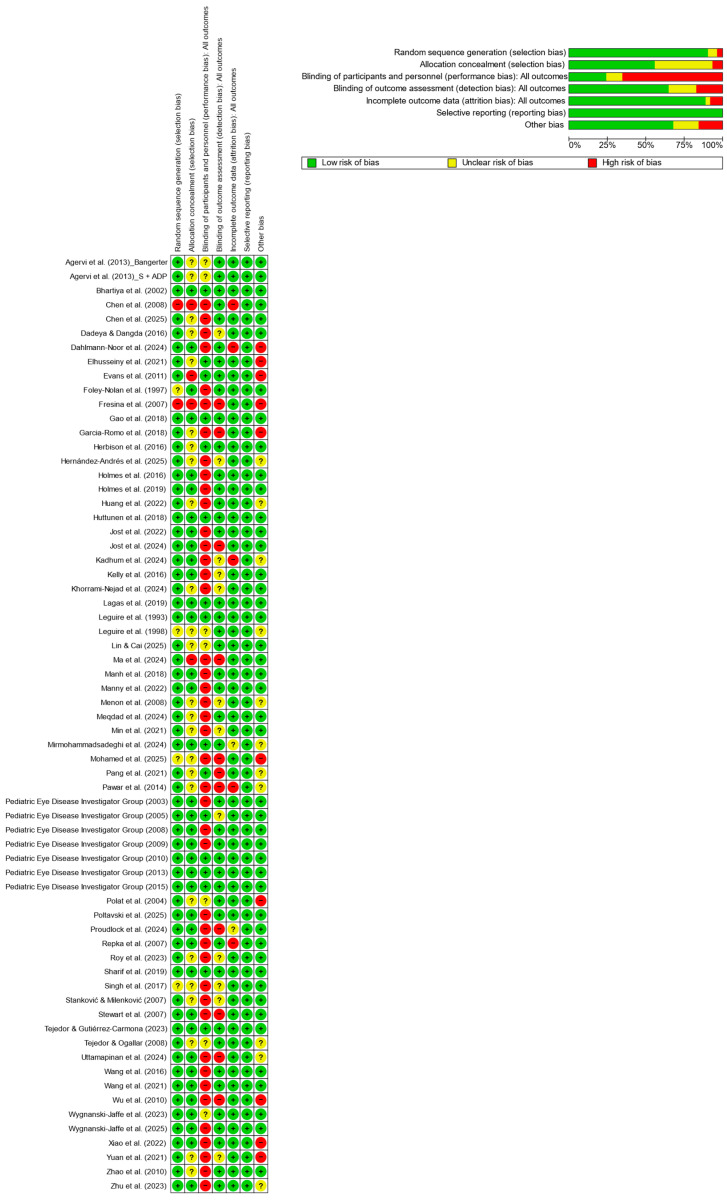
Risk of bias summary across included randomized controlled trials. The figure shows the proportion of studies rated as low (green), unclear (yellow), or high (red) risk of bias for each Cochrane Risk of Bias domain.

A domain-level summary of risk of bias judgments is presented in Figure 2. The complete study-level risk of bias matrix (study × domain) is provided in File S3, with detailed domain-specific justifications reported in File S4.

### 2.7. Assessment of Results

For continuous outcomes measured on the same scale, mean differences (MD) with 95% confidence intervals (CI) were calculated. When outcomes were reported using different measurement scales (e.g., logMAR, lines of improvement, or letters), standardized mean differences (SMDs) were used to enable pooling across studies. Mean differences and standardized mean differences were not combined within the same pooled analysis.

Visual acuity outcomes were preferentially analyzed in logMAR units. When outcomes were reported in lines or letters, values were converted to logMAR assuming 0.1 logMAR per line and 0.02 logMAR per letter, when appropriate. When conversion was not feasible or outcome scales were conceptually different, SMDs were applied.

Statistical heterogeneity was assessed using the I^2^ statistic. A fixed-effects model was applied when heterogeneity was low (I^2^ < 50%), whereas a random-effects model was used when heterogeneity was moderate or high (I^2^ ≥ 50%). For random-effects models, between-study variance (τ^2^) was estimated using the REML method.

When standard deviations were not directly reported, they were derived from standard errors, confidence intervals, *p*-values, or interquartile ranges according to Cochrane Handbook guidance. If no dispersion measure could be obtained, standard deviations were imputed from comparable studies reporting the same outcome.

Adherence outcomes and adverse events were not pooled quantitatively because of substantial heterogeneity in definitions, measurement methods, and reporting formats across studies; these outcomes are therefore summarized descriptively in Section 3.

### 2.8. Publication Bias

Potential publication bias was assessed by visually inspecting funnel plots for each primary outcome, generated with Review Manager (RevMan) version 5.4.1. Funnel plot asymmetry was interpreted as a potential indication of publication bias, suggesting the possible non-publication of smaller studies with null or inconclusive results.

### 2.9. Additional Analysis

Sensitivity analyses were conducted to evaluate the robustness of the meta-analytic results by sequentially removing studies identified as highly influential or major contributors to heterogeneity for each outcome. These analyses enabled assessment of the impact of individual studies on pooled effect estimates and helped clarify the sources of heterogeneity, especially for visual acuity and stereopsis outcomes. All sensitivity analyses were performed using Review Manager (RevMan) version 5.4.1, applying a random-effects model when moderate or high heterogeneity was detected. Additionally, the certainty of evidence for each outcome was assessed using the GRADE approach [55], considering factors such as risk of bias, inconsistency, indirectness, imprecision, and potential publication bias. All assessments were independently performed by two reviewers, with discrepancies resolved through discussion and, when necessary, consultation with a third author.

## 3. Results

### 3.1. Study Selection

A total of 3454 records were identified through database searches in PubMed (*n* = 1117), Web of Science (*n* = 965), and Scopus (*n* = 1372). In addition, six records were identified through manual screening of reference lists (Figure 1). After removal of 1487 duplicate records, 1973 records remained and were screened at the title and abstract level. Of these, 1553 records were excluded because they were not randomized controlled trials, did not include a comparison group with patching, or did not assess amblyopia treatment efficacy.

A total of 420 full-text articles were assessed for eligibility, of which 354 were excluded due to non-comparative design, inadequate or incomplete data, or an overall high risk of bias according to predefined criteria (see Section 2.6), or lack of extractable outcomes. Details of full-text exclusions and their reasons are summarized in File S5. Ultimately, 66 studies met the inclusion criteria and were included in the systematic review [21,34,35,43,56,57,58,59,60,61,62,63,64,65,66,67,68,69,70,71,72,73,74,75,76,77,78,79,80,81,82,83,84,85,86,87,88,89,90,91,92,93,94,95,96,97,98,99,100,101,102,103,104,105,106,107,108,109,110,111,112,113,114,115,116,117], of which 36 were eligible for inclusion in the meta-analysis [21,34,35,58,59,60,61,65,68,69,72,73,74,75,76,77,78,82,84,85,86,87,89,90,91,92,98,99,102,103,104,109,110,112,113,114].

Several randomized controlled trials required participants to have optimal and stable optical correction before randomization through a predefined refractive adaptation period, defined as a period of wear with appropriate refractive lenses intended to allow visual acuity stabilization and to account for improvements attributable solely to optical correction before initiating amblyopia treatment [56,57,58,65,76,85,86,90,91,92,93,94,95,96,98,109]. In contrast, other trials, particularly those evaluating digital, binocular, perceptual learning, pharmacological, or virtual reality-based interventions, did not implement a formal refractive adaptation phase but required documented stable habitual spectacle wear or visual acuity stability prior to enrollment [34,35,37,38,39,42,43,44,45,46,47,59,60,61,68,69,72,73,74,75,77,78,82,83,84,87,89,99,102,103,104,110,112,113,114,116]. Consequently, studies without a clearly defined refractive adaptation period may partially capture visual gains related to optical correction, potentially leading to an overestimation of intervention-specific treatment effects. The presence, duration, and reporting of this optical treatment phase therefore varied across studies.

### 3.2. Study Characteristics

File S6 summarizes the main characteristics of the randomized controlled trials (RCTs) included in this meta-analysis. These trials evaluated the efficacy of various amblyopia treatments in pediatric and adult populations across diverse regions, including Europe, Asia, North America, Oceania, and Africa. Sample sizes ranged from small pilot studies with 7 participants to multicenter trials enrolling up to 404 subjects. Mean participant ages varied widely, reflecting the inclusion of both pediatric and adult cohorts. Most studies included participants with anisometropic, strabismic, or mixed types of amblyopia, with a few trials focusing exclusively on one subtype.

All studies employed a randomized controlled design and compared established interventions such as patching, atropine penalization, or spectacle correction to alternative or adjunctive therapies, including dichoptic digital treatments, binocular games, virtual reality, perceptual learning, pharmacologic agents (e.g., levodopa, fluoxetine, citicoline), vision therapy, Bangerter filters, and emerging digital platforms. Treatment durations varied from short intensive interventions lasting 2 weeks to long-term regimens of up to two years.

Diagnostic criteria for amblyopia were consistent across studies, typically based on interocular visual acuity difference (commonly ≥2 or ≥3 lines), best-corrected visual acuity thresholds, and the exclusion of confounding ocular or neurologic pathology. Most studies also required a period of prior optimal refractive correction to confirm residual amblyopia before enrollment. Conflict of interest declarations were reported in nearly all studies, with only a minority disclosing potential financial or proprietary interests.

### 3.3. Outcomes

Efficacy of Dichoptic and Digital Therapies vs. Conventional Approaches

**Figure 3:**
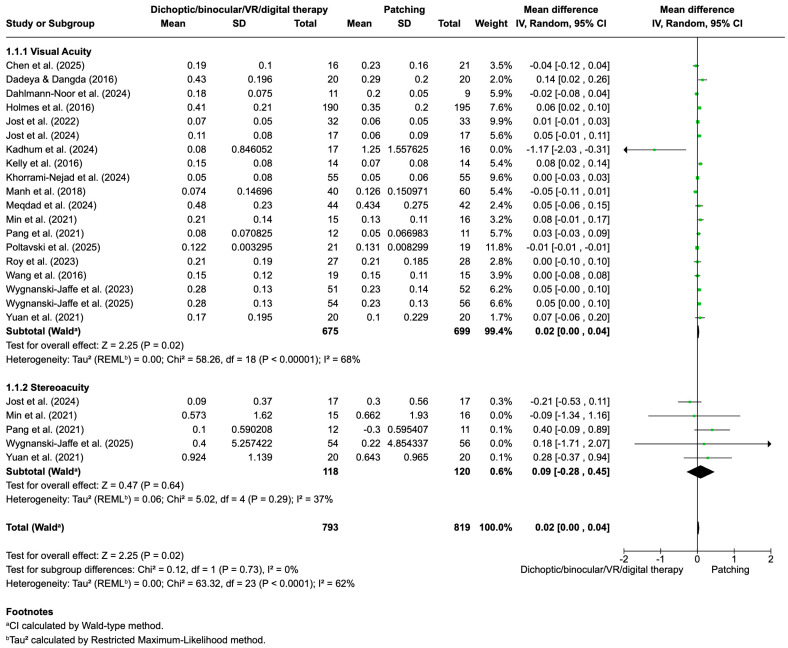
Pooled visual acuity and stereopsis outcomes in amblyopic patients treated with dichoptic/binocular/VR/digital therapy versus patching.

Figure 3 presents the pooled results for visual acuity and stereopsis outcomes comparing dichoptic/binocular/VR/digital therapy versus patching in amblyopia treatment.

Visual acuity was analyzed across 19 studies including 675 participants in the patching group and 699 in the dichoptic/binocular/VR/digital therapy group. The overall mean difference was 0.02 (95% CI: 0.00 to 0.04; *p* = 0.02), favoring dichoptic/binocular/VR/digital therapy, although the effect size was modest. Moderate heterogeneity was observed among studies (I^2^ = 68%), suggesting some variability in study designs and populations. Accordingly, a random-effects model was applied, with visual acuity outcomes preferentially analyzed in logMAR units or, when different scales were reported, pooled using standardized mean differences.

For stereopsis, five studies comprising 120 (patching group) and 118 (dichoptic/binocular/VR/digital therapy group) participants were pooled. The overall mean difference was 0.09 (95% CI: −0.28 to 0.45), indicating no statistically significant difference between groups. Heterogeneity for this outcome was lower (I^2^ = 37%); therefore, a fixed-effects model was used. When both outcomes were combined, the total pooled mean difference across 23 studies and 800 participants was 0.03 (95% CI: 0.01 to 0.05; *p* = 0.01), supporting a slight but statistically significant advantage for dichoptic/binocular/VR/digital therapy over patching. Overall heterogeneity was moderate (I^2^ = 42%). These results indicate that dichoptic/binocular/VR/digital therapy provides a small but statistically significant improvement in visual acuity compared to patching, while no significant differences were found in stereopsis outcomes. The moderate heterogeneity highlights some variation among studies, which should be considered when interpreting these findings.

The most recent studies focusing on digital binocular therapies consistently report modest or marginal benefits compared to standard patching or optical correction, particularly among older children and adolescents. For example, in Manh et al.’s [82] trial with adolescents aged 13 to <17 years, mean amblyopic eye visual acuity (VA) improved by only 3.5 letters (approximately 0.7 lines) after 16 weeks of binocular iPad game therapy, compared to 6.5 letters (1.3 lines) with 2 h of daily patching. The difference, favoring patching, was −2.7 letters (95% CI: −5.7 to 0.3; *p* = 0.082). Notably, only 13% of participants in the binocular group achieved >75% adherence, highlighting poor compliance as a likely factor in the limited effect.

This adherence challenge is mirrored in younger children. Holmes et al. [70] (children 7–12 years) and Manny et al. [83] (children 4–6 years) both evaluated the Dig Rush dichoptic game. In Holmes et al. [70], the mean improvement at 4 weeks favored binocular therapy (+1.1 lines vs. +0.6 lines for patching), but by 8 weeks, the difference narrowed (+1.3 vs. +1.2 lines), and the effect was not sustained. Similarly, in Manny et al. [83], the initial advantage of binocular therapy at 4 weeks (+0.8 lines vs. +0.5 lines) did not persist at 8 weeks. Only 47% of children in Manny et al. [83] met the ≥75% adherence threshold, reinforcing adherence as a key limitation for at-home digital therapy, regardless of age. Alternative interventions for older children and adults show different patterns. Evans et al. [63] tested Intermittent Photic Stimulation (IPS) in adolescents and adults and reported a modest gain of about one line in logMAR after six sessions. However, improvements were not maintained long-term, and the benefit was significantly greater in cases of strabismic amblyopia compared to anisometropic cases, suggesting patient selection may influence responsiveness to non-traditional approaches.

However, several recent trials have evaluated digital or binocular interventions using other comparators, such as optical correction, placebo, or standard care. Among these, some technology-supported approaches aimed at improving engagement and compliance in younger children have shown particularly promising results. Xiao et al. [118] conducted a large RCT of a dichoptic digital therapeutic (Luminopia One) for children aged 4–7 years. At 12 weeks, the treatment group improved by 1.8 lines (0.18 logMAR, 95% CI: 1.4–2.3) compared to 0.8 lines (0.08 logMAR, 95% CI: 0.4–1.3) in the glasses-only group (difference: 1.0 line; *p* = 0.0011). Importantly, median adherence to the prescribed regimen was high (88.2%), and most parents reported preferring the digital therapy over patching. A complementary approach was evaluated by Uttamapinan et al. [117], who tested a smartphone application to enhance compliance with occlusion therapy in children (mean age 7 years). At 1 month, the median compliance rate was 85% in the app group versus 64% with standard care (median difference: 22%, 95% CI: 3–48, *p* = 0.037). At 3 months, compliance decreased but remained higher in the intervention group (80% vs. 55%), though the difference was not statistically significant. Visual acuity improvement was also greater with the app at both follow-up periods (mean difference: 0.04 logMAR at 1 and 3 months), albeit gains were below one line. Finally, the study by Elhusseiny et al. [62] examined a VR-based binocular therapy prototype in older children and adults (median age 9, range 7–38), all with previous treatment failure. The intervention did not yield significant VA improvements in the amblyopic eye after 8 or 16 weeks (mean logMAR remained nearly unchanged: 0.49 at baseline vs. 0.47 at 16 weeks). However, a statistically significant improvement in stereoacuity was observed (log arcsec improved from 7.3 ± 2.0 at baseline to 6.7 ± 2.6 at 16 weeks, *p* < 0.001), suggesting some residual binocular plasticity even in older, treatment-resistant patients.

These findings are further supported by the large, multicenter BRAVO trial conducted by Gao et al. [43], which included 115 participants aged 7 to 55 years (mean age 21.5 years, most with previous patching). Participants were randomized to either an active home-based binocular video game or a placebo video game for 6 weeks. Both groups showed nearly identical mean improvements in amblyopic eye visual acuity (0.06 ± 0.12 logMAR in the active group vs. 0.07 ± 0.10 logMAR in the placebo group; adjusted mean difference: −0.02 logMAR; 95% CI: −0.06 to 0.02; *p* = 0.25). No significant differences were observed in stereoacuity, interocular suppression, or compliance between groups. Importantly, only 64% of the active group met the minimal compliance threshold (≥25% of prescribed sessions), underscoring persistent challenges with engagement, particularly among older children and adults. These results highlight that, despite theoretical advantages, current home-based binocular video game treatments do not confer additional benefit over placebo in older or previously treated amblyopic populations.

Similarly, the randomized controlled trial by Herbison et al. [67] using the I-BiT virtual reality system in children aged 4–8 years (*n* = 75) compared three arms: dichoptic video (I-BiT DVD), dichoptic interactive game, and non-dichoptic game (control). After 6 weeks, all three groups demonstrated modest but statistically significant improvements in visual acuity of approximately 0.07 logMAR in the amblyopic eye, with no significant differences between the groups. Treatment was well tolerated with high compliance (>90%) and positive acceptance among participants; however, the improvement was limited and did not differ according to the mode of dichoptic stimulation. The authors noted that the high proportion of previously treated and strabismic amblyopia may have limited the observed effect, and that short treatment duration was also a constraint. These findings reinforce the conclusion that, while dichoptic or technology-based interventions are safe and acceptable, their efficacy remains modest and comparable to standard care, particularly in populations with prior amblyopia therapy or more complex amblyopia subtypes.

Atropine vs. Conventional Approaches

**Figure 4:**
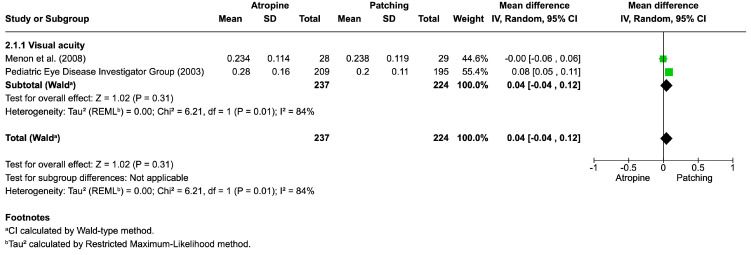
Pooled analysis of visual acuity outcomes comparing atropine versus patching in amblyopia treatment.

Figure 4 presents the pooled results for visual acuity comparing atropine versus patching in the treatment of amblyopia. Visual acuity outcomes were synthesized from two randomized controlled trials, including a total of 237 participants in the atropine group and 224 in the patching group. The overall mean difference in visual acuity was 0.04 (95% CI: −0.04 to 0.12; *p* = 0.31), indicating no statistically significant difference between atropine and patching. Substantial heterogeneity was observed among the included studies (I^2^ = 84%); therefore, a random-effects model was applied.

At the individual study level, the Pediatric Eye Disease Investigator Group (2003) [22] reported a small mean difference of 0.08 (95% CI: 0.05 to 0.11) in favor of patching, whereas Menon et al. [85] found virtually no difference between the two treatments (mean difference = −0.00; 95% CI: −0.06 to 0.06). When data were pooled, the results did not demonstrate superiority for either treatment.

Recent evidence directly comparing atropine penalization and patching as primary treatments for amblyopia demonstrates equivalent efficacy in improving visual acuity, with notable differences in compliance and acceptability. In the randomized trial by Foley-Nolan et al. [64], 36 treatment-naive children with amblyopia (mean age 5.5 years) were assigned to either daily atropine 1% drops or traditional patching. Both groups showed significant improvements in the amblyopic eye: the geometric mean acuity improved from 6/50 to 6/11 in the atropine group and from 6/60 to 6/19 in the patching group (both *p* < 0.001). Importantly, compliance was markedly higher in the atropine group (94% vs. 55%), and patient acceptance was superior, with no cases of occlusion amblyopia reported in either group. The authors concluded that atropine penalization is as effective as occlusion therapy and may be preferable due to better compliance and ease of monitoring treatment adherence.

These findings are corroborated and extended by the larger, multicenter PEDIG randomized trial [93], which evaluated 193 children aged 7 to 12 years with moderate amblyopia (20/40–20/100) assigned to either weekend atropine or two hours per day of patching. After 17 weeks, mean visual acuity improved by 7.6 letters in the atropine group and 8.6 letters in the patching group, with an adjusted mean difference of just 1.2 letters (95% CI: −0.7 to +3.1), meeting strict criteria for equivalence. About 20% of subjects in both groups achieved 20/25 or better vision in the amblyopic eye, and improvement was similar regardless of baseline acuity, cause of amblyopia, or prior treatment. While both treatments were well tolerated, quality-of-life assessment with the Amblyopia Treatment Index favored atropine, particularly regarding compliance and social stigma, despite similar adverse effect profiles. Stereoacuity improved similarly in both groups, and no cases of reverse amblyopia were detected. These results reinforce the conclusion that atropine penalization is a robust alternative to patching, especially in older children, offering comparable clinical benefits with potentially higher acceptability and adherence for families.

Further supporting the safety and refractive neutrality of atropine penalization, Repka et al. [101] conducted a large-scale randomized clinical trial assessing changes in refractive error associated with unilateral atropine versus occlusion. Among 282 children aged 3 to <7 years treated for up to two years, mean change in the spherical equivalent refractive error of the sound eye was minimal and similar between atropine (+0.10 D) and patching (+0.08 D) groups, with no significant differences detected. These findings indicate that long-term atropine use does not induce asymmetric refractive changes compared to occlusion, alleviating concerns about refractive development in hypermetropic children undergoing penalization therapy.

Other studies have compared atropine penalization with alternative interventions, such as optical penalization or adjunctive treatments. One such study is the prospective trial by Tejedor et al. [107], which evaluated the effectiveness of atropine versus optical penalization using overplus lenses in 63 children (aged 2–10 years) with moderate or mild amblyopia. After six months, average improvement in amblyopic eye visual acuity was significantly greater in the atropine group (3.4 logMAR lines) compared to the optical penalization group (1.8 lines; *p* = 0.01). Similarly, the reduction in interocular acuity difference was larger with atropine (2.8 vs. 1.3 lines), and although both groups achieved comparable stereoacuity outcomes, a higher proportion of children gained at least three lines with atropine. These results support the preferential use of atropine penalization over optical defocus in this population.

In the context of optimizing visual rehabilitation for specific subtypes, the addition of bifocals or plano lenses has also been investigated. A proof-of-concept randomized trial by Tejedor and Gutiérrez-Carmona [108] evaluated the addition of a bifocal lens in the amblyopic eye for children with hyperopic anisometropic amblyopia treated with atropine. At six months, improvement in amblyopic eye visual acuity was significantly greater in the atropine plus bifocal group (3.3 ± 0.9 logMAR lines) than with atropine alone (2.6 ± 0.8 lines; *p* = 0.04), while gains in stereoacuity and contrast sensitivity were similar. The bifocal approach addresses the accommodative lag commonly present in anisometropic amblyopia and may further enhance visual outcomes in selected cases. Additionally, a recent randomized clinical trial by the PEDIG [97] explored whether augmenting ongoing atropine penalization with a plano lens over the fellow eye could yield further improvements in children with stable residual amblyopia after initial atropine treatment. Among 73 children aged 3 to <8 years, those randomized to atropine plus plano lens demonstrated a greater mean improvement in amblyopic eye visual acuity at 10 weeks (1.1 lines) compared to atropine alone (0.6 lines), although the difference was not statistically significant (adjusted mean difference +0.5 lines, 95% CI: –0.1 to +1.2). At the visit with the best-recorded outcome, the cumulative gain favored the plano group (1.9 vs. 0.8 lines). While the benefit of plano lens augmentation did not reach statistical significance, these results indicate a potential role for combining optical blur with pharmacological penalization, particularly in children with incomplete response to atropine alone.

Adherence and Adverse Events

Adherence was heterogeneously reported across trials and assessed using different metrics, including prescribed patching hours, percentage of completed digital sessions, or device-recorded usage. In patching studies, reported adherence typically ranged from approximately 50% to 85% of the prescribed dose, with objectively monitored studies showing lower effective patching times than prescribed.

For digital and binocular interventions, adherence varied widely: several home-based interventions reported that fewer than 50% of participants achieved ≥75% of prescribed sessions, particularly among older children and adolescents, whereas supervised or technology-assisted digital therapies reported substantially higher adherence, frequently exceeding 80%.

Adverse events were uncommon and generally mild across all interventions, including transient skin irritation with patching, mild photophobia with atropine, and short-lasting visual discomfort or headache with digital or VR-based therapies. No serious or persistent adverse events were reported.

Pharmacological Adjuvant (Levodopa, CDP-choline, etc.) + Patching vs. Patching

**Figure 5:**
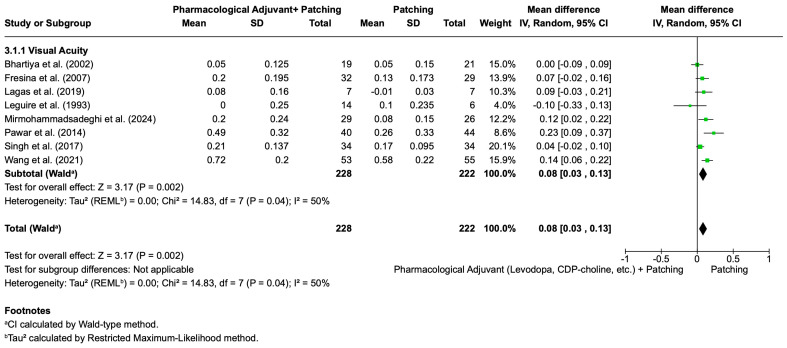
Visual acuity outcomes: pharmacological adjuvant therapy plus patching versus patching alone.

Figure 5 presents the pooled results for visual acuity improvement comparing pharmacological adjuvant therapy (levodopa, CDP-choline, etc.) plus patching versus patching alone. A total of eight studies were included, with 228 participants in the pharmacological adjuvant plus patching group and 222 in the patching group. The overall mean difference was 0.08 logMAR (95% CI: 0.03 to 0.13; *p* = 0.002) in favor of pharmacological adjuvant plus patching. Moderate heterogeneity was observed (I^2^ = 50%), and results were therefore estimated using a random-effects model.

Recent randomized trials have explored pharmacologic and combined penalization strategies to optimize amblyopia treatment outcomes. The PEDIG multicenter trial [94] directly compared weekend atropine penalization plus a plano lens versus weekend atropine alone in 180 children (mean age ~5 years) with moderate amblyopia. After 18 weeks, both groups showed substantial improvements in amblyopic eye visual acuity: the atropine plus plano lens group improved by an average of 2.8 lines and the atropine-only group by 2.4 lines (adjusted mean difference, 0.3 lines; 95% CI: −0.2 to 0.8). The proportion of children achieving 20/25 or better vision in the amblyopic eye was higher in the atropine plus plano lens group (40% vs. 29%, *p* = 0.03), but no significant differences were found for three-line improvement. Importantly, a greater incidence of temporary reduced sound eye acuity was observed in the plano lens group, but no persistent reverse amblyopia occurred. These results indicate that augmentation of atropine penalization with optical blur via a plano lens does not substantially enhance amblyopic eye visual acuity over atropine alone as first-line treatment, though it may increase the proportion achieving normative acuity.

Complementing these findings, Leguire et al. [79] conducted a randomized trial in older, previously treated children with amblyopia (mean age ~9 years), comparing the effect of oral levodopa–carbidopa with and without part-time occlusion (3 h/day) over seven weeks. Both groups experienced significant improvement in amblyopic eye visual acuity, but the combination of levodopa–carbidopa and occlusion yielded a significantly greater benefit (from 20/116 to 20/76, +2.1 lines, *p* < 0.001) compared to levodopa–carbidopa alone (from 20/90 to 20/73, +0.8 lines, *p* < 0.01; between-group difference, *p* = 0.01). The occlusion group also showed greater reduction in interocular difference and significant gains in contrast sensitivity, with improvements maintained at four-week follow-up. Adverse effects were minimal and similar across groups.

Patching regimens

Recent randomized evidence directly comparing regimens of occlusion and refractive adaptation in amblyopia highlights important nuances regarding efficacy, compliance, and the potential for individualized treatment strategies. In a prospective, randomized study by Agervi et al. [57], 40 children (mean age 4.3 years) with strabismic or mixed amblyopia were assigned to spectacles plus alternate-day patching (≥8 h) or spectacles plus daily patching (≥8 h, 6 days per week). After two years, the median improvement in amblyopic eye visual acuity was significantly greater in the alternate-day group (0.8 logMAR) than in the daily patching group (0.6 logMAR, *p* = 0.045). However, the final median acuity did not differ between groups (0.0 vs. 0.1 logMAR). Both protocols were well tolerated, with improvements in binocularity and no significant adverse events, supporting alternate-day patching as an effective and potentially more manageable regimen for strabismic amblyopia.

Expanding on the role of refractive adaptation, the large multicenter EuPatch trial by Proudlock et al. [100] investigated whether an extended period of optical correction before intensive patching confers additional benefit compared to early initiation of patching. In this randomized controlled trial involving 334 treatment-naive children aged 3–8 years with amblyopia of varying etiology, participants were allocated to either 18 weeks of full-time glasses wear prior to patching (EOT) or only 3 weeks of glasses before starting intensive patching (10 h/day, 6 days/week). After 12 weeks of patching, the rate of successful treatment (≤0.20 logMAR interocular difference) was higher in the early patching group (67%) compared to the EOT group (54%; *p* = 0.019). Notably, the advantage of early patching was most apparent in older children and those with severe amblyopia or large interocular refractive differences, while younger children with mild amblyopia responded well to extended wearing of glasses alone. These results suggest that earlier initiation of patching may accelerate visual gains and improve overall outcomes in most cases, challenging current guidelines that advocate for prolonged refractive adaptation prior to occlusion.

The intensity and structure of occlusion regimens remain a topic of debate, particularly in the management of profound strabismic amblyopia in older children. In a prospective randomized study by Stanković and Milenković [105], 53 children over 5 years old with severe amblyopia (VA ≤ 0.4, post-adaptation) were randomized to either continuous full-time occlusion of the sound eye (24 h/day) or an alternating regimen (full-time occlusion of the sound eye for ‘one day more than the child’s age’, followed by one day of occluding the amblyopic eye). Both approaches yielded comparable rates of cure (41%) and significant improvements in visual acuity, with greater gains in younger participants. While continuous occlusion was associated with occasional reversible occlusion amblyopia in the fellow eye, both regimens were generally well tolerated. The authors concluded that, for older children with profound amblyopia, intensive patching, whether continuous or alternating, remains effective, but treatment must be individualized to minimize risks.

Regarding the optimal daily duration of occlusion, Stewart et al. [106] conducted a randomized trial with 97 children (mean age, 5.6 years) comparing prescribed patching of 6 versus 12 h daily, following a standardized period of refractive adaptation (18 weeks). Objectively monitored compliance revealed that the actual dose received was lower than prescribed in both groups (mean 4.2 vs. 6.2 h/day), yet visual outcomes were similar: mean improvement in amblyopic eye acuity was 0.26 logMAR in the 6 h group and 0.24 logMAR in the 12 h group (*p* = 0.64). Children under 4 years required the least occlusion for effective treatment, and neither amblyopia type nor patching duration significantly affected outcomes. These findings reinforce the conclusion that substantial doses of patching are as effective as maximal regimens, highlighting the importance of realistic, family-friendly protocols to optimize adherence.

Most recently, the PEDIG [96] conducted a large multicenter randomized trial to evaluate the benefit of increasing patching intensity in children aged 3 to <8 years with residual stable amblyopia after at least 12 weeks of 2 h daily patching. In this study, 169 participants were randomized to either continue 2 h per day or increase to 6 h per day of patching. After 10 weeks, mean improvement in amblyopic eye visual acuity was significantly greater in the 6 h group (1.2 lines) compared to the 2 h group (0.5 lines), with an adjusted mean difference of 0.6 lines (95% CI: 0.3–1.0; *p* = 0.002). Moreover, 40% of children in the 6 h group improved by 2 or more lines, versus 18% in the 2 h group (*p* = 0.003). These results support a stepwise approach in which patching dose can be increased if visual improvement plateaus on a lower regimen, while also highlighting that a proportion of children may continue to improve with 2 h per day even after apparent stabilization.

Alternative/additive treatments vs. Patching

Recent randomized and comparative studies have evaluated whether novel adjunctive or alternative therapies to patching can enhance visual outcomes or improve compliance in the management of amblyopia, especially in cases refractory to conventional occlusion. Wu et al. [111] conducted a pilot randomized trial in children aged 4 to 17 years with refractory amblyopia, comparing 30 min per day of patching alone to patching combined with telescopic magnification for the amblyopic eye. After 17 weeks, both groups demonstrated significant improvement in visual acuity, but the additional use of telescopic magnification did not confer any significant benefit over patching alone (mean gain 0.14 logMAR vs. 0.06 logMAR; *p* = 0.11). The study concluded that occlusion and penalization remain the gold standard for amblyopia treatment, with novel optical devices requiring further evidence before adoption.

A different line of research has focused on acupuncture as either an adjunct or alternative to patching, particularly in anisometropic amblyopia. In a large randomized controlled trial, Zhao et al. [115] assigned 88 children (7–12 years) to receive either 2 h daily patching or five weekly acupuncture sessions, all following a period of optimized spectacle correction. Both groups achieved significant improvements in best-corrected visual acuity (BSCVA) at 15 weeks, but the acupuncture group showed a statistically greater mean improvement (2.27 vs. 1.83 lines; adjusted mean difference 0.049 logMAR, *p* = 0.03) and higher rates of amblyopia resolution (41.5% vs. 16.7%). The findings support acupuncture as a potentially equivalent and even superior alternative to patching in selected older children.

These results are further corroborated by Ma et al. [81], who examined the efficacy and neurobiological effects of combining acupuncture with conventional therapy in 76 children with anisometropic amblyopia. Patients were randomized to receive conventional treatment (patching plus visual stimulation) or the same regimen plus acupuncture at specific ocular and extraocular points. The combination group achieved higher rates of clinical response (86.1% vs. 65.8%, *p* < 0.05) and greater improvements in best-corrected visual acuity after four weeks. Functional MRI analyses suggested that acupuncture augmented regional brain activity in visual “what” pathway areas, including the right inferior occipital gyrus and fusiform gyrus, providing mechanistic insight into its therapeutic benefit.

Another alternative approach, syntonic phototherapy, was assessed by Mohamed et al. [88] in a randomized trial of 40 patients (mean age 14.4 years) with refractive amblyopia, comparing partial occlusion to a protocol of syntonic light stimulation. While both groups improved, the syntonic phototherapy group showed significantly greater gains in both uncorrected and best-corrected visual acuity, as well as in measures of visual function and field, suggesting this modality may offer a promising adjunct or alternative to occlusion, particularly in older children and adults.

The search for more tolerable or acceptable occlusive modalities has also included prosthetic occluding contact lenses (OCLs). Garcia-Romo et al. [66] conducted a prospective case series in amblyopic adults (mean age 40 years), randomly assigned to OCLs or standard patching for at least 4 h per day. Both groups demonstrated significant visual acuity improvement after 1.5 months, but OCL users reported higher vision-related quality of life scores at 12 months, indicating better acceptability and potentially improved adherence.

Recent technological innovations have sought to leverage advances in perceptual learning and binocular integration for amblyopia therapy. In a randomized trial, Huang et al. [71] evaluated asynchronous binocular visual stimulation based on spike-timing dependent plasticity (STDP) principles versus traditional patching in children with anisometropic amblyopia. Only 10.5 h of asynchronous conditioning (e.g., via 3D movies with controlled timing) over less than three weeks achieved treatment success at a rate and durability significantly greater than patching alone, with benefits maintained for up to two years. This novel binocular intervention was reported to be about 50 times more efficient than patching and was highly popular among children, further highlighting the importance of acceptability and engagement.

Bangerter filter and optical/pharmacological penalization

Recent randomized controlled trials have critically evaluated the efficacy and acceptability of Bangerter filters and optical or pharmacological penalization compared to traditional patching as primary therapies for amblyopia. In a pivotal multicenter trial by the PEDIG [95], 186 children with moderate amblyopia (20/40 to 20/80) aged 3 to <10 years were randomized to receive either daily patching or a Bangerter filter applied to the spectacle lens of the fellow eye. After 24 weeks, mean visual acuity improvement in the amblyopic eye was 1.9 lines in the Bangerter group and 2.3 lines in the patching group, a difference of less than half a line (adjusted mean difference: 0.38 line, 95% CI: −0.06 to +0.83), which did not meet the predefined non-inferiority margin, but patching was not statistically superior. Importantly, the proportions of children achieving improvements of ≥3 lines (Bangerter 38%, patching 35%) or attaining 20/25 or better acuity (36% vs. 31%) were similar. Treatment burden, as assessed by the Amblyopia Treatment Index, was significantly lower for the Bangerter filter, especially regarding compliance and social stigma, making it a more acceptable option for many families. Stereoacuity outcomes and adverse event rates were also similar between groups.

These findings are further supported by Agervi et al. [56], who conducted a two-year randomized trial in 80 children (mean age 4.4 years) with anisometropic amblyopia, comparing spectacle correction alone versus spectacles combined with a full-time Bangerter filter on the fellow eye. While Bangerter filters yielded a more rapid improvement in visual acuity during the first months of treatment, by two years, both groups had achieved comparable median gains (0.4 logMAR in both), with a final median visual acuity of 0.0 logMAR in the amblyopic eye. Amblyopia resolved in nearly all cases, and improvements in binocular function and refractive error were observed in both arms. These results confirm the long-term stability and equivalence of outcomes between Bangerter filter penalization and conventional optical correction for anisometropic amblyopia.

Most recently, Zhu et al. [116] evaluated the effectiveness of a combined binocular therapy (Bangerter filter plus immersive 3D movie viewing) together with part-time patching, versus patching alone, in older children (5–12 years) with residual amblyopia or poor response to standard occlusion. In this randomized trial, after six weeks, mean visual acuity improvement in the amblyopic eye was 0.17 logMAR in the combined group, compared to only 0.05 logMAR in the patching group (mean difference: 0.13 logMAR [1.3 lines]; 95% CI: 0.08–0.17; *p* < 0.01). Furthermore, only the combined group demonstrated a significant gain in stereoacuity (mean improvement: 0.47 log arcsec), with nearly half of the patients achieving at least a two-line improvement in visual acuity and normalization of binocular function scores. This laboratory-based approach, with high treatment compliance, highlights the potential benefit of engaging, immersive binocular strategies, especially for children with previous poor compliance or limited response to conventional patching.

Other treatments

**Figure 6:**
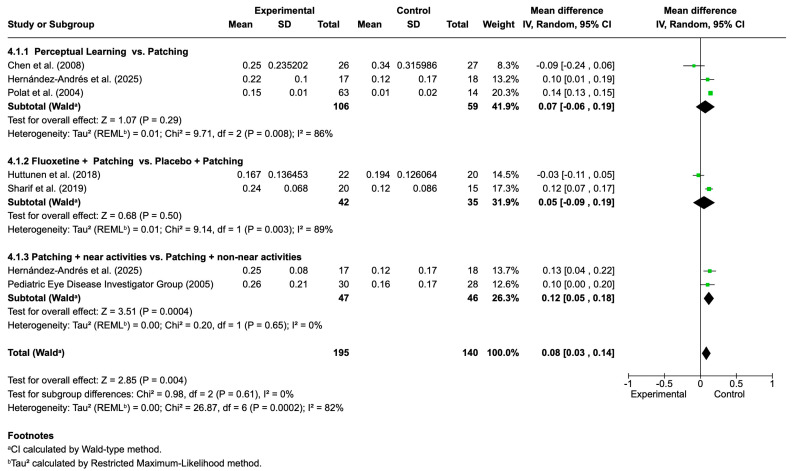
Pooled mean difference in visual acuity improvement for experimental interventions versus standard patching in amblyopia.

Figure 6 presents the pooled results for visual acuity outcomes comparing several adjunctive or alternative interventions to patching in the treatment of amblyopia. The analysis was divided into three subgroups: (1) perceptual learning versus patching, (2) fluoxetine plus patching versus placebo plus patching, and (3) patching combined with near activities versus patching with non-near activities.

Across all included studies (*n* = 7), a total of 195 participants were allocated to the experimental interventions and 140 to the control (patching) group. The overall mean difference in visual acuity improvement favored the experimental interventions (MD = 0.08; 95% CI: 0.03 to 0.14; *p* = 0.004), indicating a statistically significant but modest benefit over patching alone. Considerable heterogeneity was observed among studies (I^2^ = 82%), and pooled estimates were therefore derived using a random-effects model.

Subgroup analyses revealed that perceptual learning interventions yielded a small and non-significant improvement over patching alone (mean difference 0.07; 95% CI: −0.06 to 0.19; I^2^ = 86%). For fluoxetine combined with occlusion versus placebo plus occlusion, the pooled mean difference was also non-significant (0.05; 95% CI: −0.09 to 0.19; I^2^ = 89%). For the comparison between patching combined with near activities and patching with non-near activities, the pooled estimate showed a small statistically significant difference favoring near activities (mean difference 0.12; 95% CI: 0.05 to 0.18; I^2^ = 0%). However, the magnitude of this effect was modest, and the evidence should be interpreted cautiously, as the observed improvement is small and may reflect differences in study design, sample characteristics, or intervention intensity rather than a consistent clinical advantage. Recently, novel neurostimulation-based approaches have also shown promise as adjuncts to traditional amblyopia therapies. Lin and Cai [80] conducted a randomized controlled trial in 148 adults with anisometropic amblyopia to evaluate the effects of repetitive transcranial magnetic stimulation (rTMS) combined with intraocular collamer lens (ICL) implantation versus ICL alone. After three months, both groups showed significant improvements in best-corrected visual acuity (BCVA) and random dot stereopsis, but the combined ICL + rTMS group achieved significantly greater gains (ΔBCVA: 0.27 vs. 0.10; *p* < 0.05). Moreover, the ICL + rTMS group demonstrated a higher proportion of participants achieving fine (second-order) stereopsis and greater reduction in stereo disparity compared to ICL alone. Functional MRI analyses revealed that rTMS promoted distinct patterns of neuroplasticity, with enhanced activity in the occipital lobe and reduced compensatory activation in the frontal and temporal lobes. These findings support rTMS as a non-invasive and effective strategy to enhance visual cortical neuroplasticity, leading to improved visual function and binocular outcomes in adults with amblyopia, an age group typically considered refractory to conventional therapy.

### 3.4. Sensitivity Analysis

**Figure 7:**
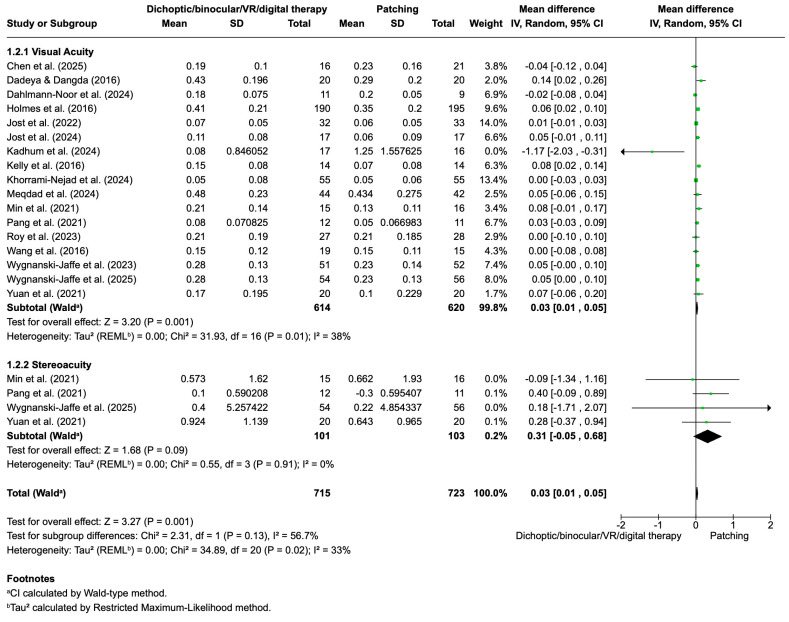
Sensitivity analysis of dichoptic/binocular/VR/digital therapy versus patching after exclusion of influential studies.

A sensitivity analysis was conducted by excluding specific studies identified as major contributors to heterogeneity in the pooled analysis of visual acuity and stereopsis outcomes (Figure 7). For visual acuity, the removal of Manh et al. [82] and Poltavski et al. [99] led to a reduction in heterogeneity from 68% to 38%, while the pooled mean difference remained statistically significant at 0.03 (95% CI: 0.01 to 0.05; *p* = 0.001), still favoring dichoptic/binocular/VR/digital therapy over patching. This indicates that the observed benefit in visual acuity is robust and not driven by these outlier studies.

For stereopsis, exclusion of Jost et al. [74] further decreased heterogeneity from 37% to 0%, resulting in a pooled mean difference of 0.31 (95% CI: −0.05 to 0.68; *p* = 0.09), which, although in favor of digital therapies, did not reach statistical significance. The overall heterogeneity across both outcomes was reduced to 33% after these exclusions, supporting the consistency and reliability of the meta-analytic findings.

**Figure 8:**
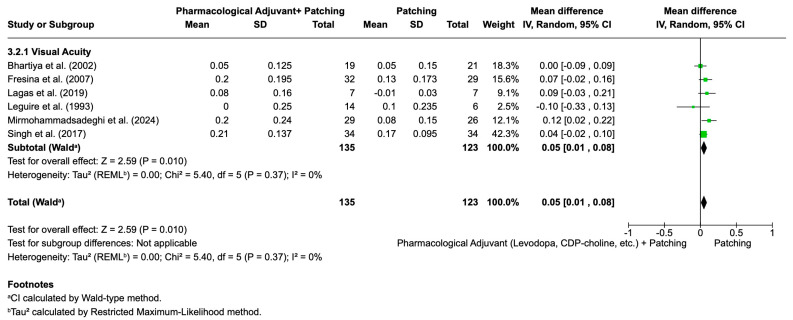
Sensitivity analysis of pharmacological adjuvant therapy plus patching versus patching alone after exclusion of influential studies.

Another sensitivity analysis was conducted by excluding the studies identified as primary contributors to heterogeneity, Pawar et al. [90] and Wang et al. [110], from the pooled analysis of visual acuity improvement with pharmacological adjuvant therapy plus patching versus patching alone (Figure 8). After removing these studies, the meta-analysis included six randomized controlled trials with 135 participants in the pharmacological adjuvant plus patching group and 123 in the patching group. The recalculated mean difference was 0.05 logMAR (95% CI: 0.01 to 0.08; *p* = 0.010), still favoring pharmacological adjuvant therapy, though the effect size was slightly reduced compared to the initial pooled result (0.08 logMAR; 95% CI: 0.03 to 0.13; *p* = 0.002). Importantly, heterogeneity was eliminated (I^2^ = 0%) in the sensitivity analysis, indicating complete consistency across the remaining studies.

### 3.5. Publication Bias

**Figure 9:**
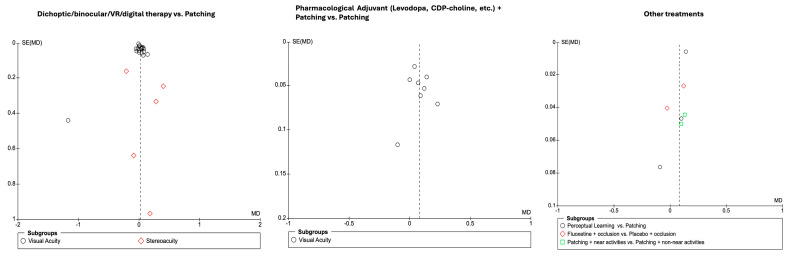
Assessment of publication bias.

Publication bias was assessed using funnel plots for all primary outcome domains in the meta-analysis, including visual acuity outcomes across key treatment comparisons. Visual inspection of the funnel plots (Figure 9) revealed varying degrees of asymmetry between intervention groups. For the comparison between dichoptic/binocular/VR/digital therapy and patching, there was noticeable asymmetry, particularly among studies reporting stereopsis outcomes, suggesting the possibility of small-study effects or publication bias in this domain. In contrast, the funnel plot for pharmacological adjuvant therapy (levodopa, CDP-choline, etc.) plus patching versus patching alone demonstrated a more symmetrical distribution, with little evidence of publication bias for visual acuity outcomes. Finally, subgroup analyses of visual acuity outcomes across perceptual learning, fluoxetine plus occlusion, and near activities versus non-near activities showed a generally symmetric distribution, further supporting the robustness of the findings in these subgroups. Overall, the degree of funnel plot asymmetry varied by outcome and intervention group, with more pronounced effects observed in outcome domains characterized by higher heterogeneity or smaller sample sizes.

### 3.6. GRADE

The GRADE (Grading of Recommendations, Assessment, Development and Evaluation) summary of findings for all major comparisons is presented in Table 2. Overall, the certainty of evidence ranged from low to moderate across outcomes. Moderate-certainty evidence supported the equivalence between atropine penalization and patching, as well as the modest additional benefit of dichoptic/binocular/VR/digital therapies and pharmacological adjuvants when combined with patching. Low-certainty evidence was observed for perceptual learning, fluoxetine as an adjunct to patching, and patching combined with near activities. Downgrading of certainty was primarily driven by substantial statistical and clinical heterogeneity and, for some comparisons, by imprecision related to small numbers of trials and wide confidence intervals. All outcomes were considered critical for clinical decision-making.

## 4. Discussion

In this meta-analysis, dichoptic, binocular, virtual reality, and digital therapies were associated with a small but statistically significant improvement in visual acuity compared with occlusion. From a clinical standpoint, a change of approximately 0.1 logMAR (equivalent to one line of visual acuity) is commonly considered the minimum threshold for a clinically meaningful improvement in amblyopia. When interpreted against this benchmark, the pooled effects observed for digital/binocular therapies (≈0.02 logMAR) and for pharmacological adjuvants combined with patching (≈0.08 logMAR) are modest and, in most cases, fall below or only approach clinical relevance. Accordingly, these statistically significant differences should be interpreted cautiously. Importantly, these effects should be interpreted as incremental to appropriate optical correction, which was required or confirmed in most included trials and remains the first-line treatment for amblyopia. The certainty of evidence, assessed using the GRADE framework, ranged from low to moderate across outcomes, further supporting a pragmatic interpretation that prioritizes adherence, tolerability, and patient-centered factors over small numerical gains in visual acuity. No significant differences were observed in stereopsis outcomes, and moderate heterogeneity across studies reflects variability in study design and populations. These results align partially with Aljohani [119], who reported that while digital therapies such as Luminopia or CureSight show preliminary improvements in efficacy and adherence, they do not consistently outperform conventional methods, particularly in older children, and that adherence remains a key limiting factor. In our data, the initial gains reported in trials such as those by Holmes [70] or Manny [83] were not always sustained, likely due to loss of interest, a trend also reflected by Kadhum et al. [120], who found that nearly half of the children discontinued VR therapy before completion because of a lack of motivation or comprehension difficulties in those under 5.5 years of age. The review by Wallace et al. [18] based on objective monitoring using occlusion dose monitors (ODMs), reported a mean adherence below 50%, supporting the idea that insufficient adherence is a cross-cutting issue for all types of therapy. Strategies such as the wordless educational cartoon used by Tjiam et al. [121] have proven effective in improving adherence in populations with language barriers, reinforcing the notion that motivation and educational support are critical elements. Moreover, comparisons with the evidence from Roda et al. [122] Tsani et al. [123] and Tang et al. [124] confirm that current binocular therapies do not replace occlusion as the first-line treatment, though they may serve as useful adjuncts, and that baseline visual acuity and training duration have more influence on outcomes than the treatment modality itself. This may be because, although digital therapies provide active binocular stimulation, their effectiveness is highly dependent on consistent training and the child’s attention span, which introduces variability and limits their advantage over more standardized passive methods such as patching.

Regarding pharmacological penalization, our meta-analysis shows that atropine is equivalent to occlusion in visual efficacy, with potential advantages in acceptability and adherence, in agreement with the 15-year PEDIG [125] follow-up, which demonstrated stable visual outcomes without differences between treatments. Findings by van Minderhout et al. [30] add that factors such as skin pigmentation and behavioral responses (crying) can influence cycloplegic efficacy, which might explain interindividual variations in the included trials. This aspect, rarely evaluated systematically, could be relevant in clinical practice when personalizing treatment choices. The similarity in results between atropine and patching is likely explained by the fact that both interventions share the same functional mechanism, forcing use of the amblyopic eye, although they differ in the route of application, which affects tolerance and adherence more than physiological efficacy.

In the analysis of pharmacological adjuvants such as levodopa or CDP-choline combined with occlusion, we observed a modest but consistent additional benefit in visual acuity, even after sensitivity analyses removing higher-heterogeneity studies. This finding is consistent with the trial by Leguire et al. [79], which showed greater gains with levodopa–carbidopa plus occlusion compared to monotherapy, and is complemented by results such as those from Tuna et al. [126], where theta-burst transcranial magnetic stimulation in adults improved visual acuity, stereopsis, and binocular balance, suggesting that interventions enhancing cortical plasticity could increase the effectiveness of conventional treatments, even at ages typically considered less responsive. These differences are likely because pharmacological adjuvants or neurostimulators act on visual neuroplasticity, enhancing the system’s capacity to respond to therapeutic stimulus, although the absolute impact is moderate due to individual variability and the limited duration of intervention.

Regarding patching regimens, our results indicate that modifications such as increasing daily hours in cases of stable response or alternating-day occlusion can offer similar benefits to more intensive regimens, with potential improvements in manageability. This is consistent with studies such as one by Agervi et al. [57], who found that alternate-day occlusion achieved equivalent results to daily occlusion in strabismic amblyopia, and with the EuPatch trial by Proudlock et al. [100], which questioned prolonged optical adaptation before starting occlusion, particularly in severe cases or older children. Trials such as PEDIG’s occlusion intensification study [96] also support the conclusion that stepwise dose increases can be useful if improvement plateaus on lower regimens. The absence of relevant differences suggests that the therapeutic visual stimulus reaches an efficacy threshold that does not necessarily increase with more hours, supporting more flexible protocols that improve adherence without sacrificing outcomes.

Within the category of alternative or additive treatments to occlusion, our analysis confirms that interventions such as acupuncture or optical stimulation can provide additional benefits in certain contexts. For example, Zhao et al. [115] reported higher resolution rates in anisometropic amblyopia compared to occlusion alone, while Ma et al. [81] demonstrated visual improvements and neurofunctional changes on fMRI when combining acupuncture with conventional therapy. Other approaches, such as syntonic phototherapy [88] or occlusive contact lenses [66], have also demonstrated improvements, particularly in acceptability, although the evidence remains limited. These benefits may be due to activation of complementary visual pathways or a motivational effect associated with the novelty of the treatment. However, the lack of standardized protocols and the small number of studies prevent definitive conclusions.

With respect to Bangerter filters and optical or pharmacological penalization, the meta-analysis indicates that visual acuity performance is comparable to occlusion, but with lower perceived treatment burden and better acceptance, as reflected in the PEDIG [95] and Agervi et al. [56] trials. Furthermore, studies such as Zhu et al. [116] suggest that combining Bangerter filters with immersive binocular therapies and partial occlusion may improve both visual acuity and stereopsis compared to occlusion alone, aligning with our interpretation that hybrid strategies may maximize benefits. The equivalence in efficacy is probably because the filters achieve a sufficient level of penalization to stimulate the amblyopic eye, but by allowing some binocular use, they reduce psychological and social impact, thereby improving adherence and patient quality of life.

Our analysis of perceptual learning revealed modest improvements, aligning with Huang et al.’s [71] findings that the amblyopic adult visual system is broader generalization than the normal visual system, suggesting potential for more specific and intensive protocols. In contrast, evidence from studies on fluoxetine combined with occlusion, included in this review, showed no significant advantage over placebo plus occlusion, indicating limited clinical benefit without optimized treatment regimens. Conversely, combining occlusion with near activities showed a significant benefit compared to non-near activities, consistent with the hypothesis that tasks requiring sustained fixation and accommodation can enhance plasticity and accelerate visual recovery. These differences may reflect the fact that perceptual learning and near tasks provide active, repetitive visual stimulus that facilitates consolidation of visual acuity improvements, whereas pharmacological interventions such as fluoxetine require an optimized training environment to demonstrate additional benefits.

The visual improvement observed in our analysis was maintained for both distance and near vision, in line with the results of Christoff et al. [127], who found no systematic differences between the two distances in moderate amblyopia, suggesting that therapeutic benefits are transferable to different visual demands. Overall, this broad comparison with the literature shows consistency in several key points: the equivalence between atropine and occlusion; the complementary but non-substitutive role of digital and binocular therapies; the modest but real benefit of some pharmacological adjuvants; the possibility of adapting occlusion regimens without loss of efficacy; and the relevance of integrating stimulating activities or penalizing filters to improve acceptability and adherence, which remains the main determinant of therapeutic success in amblyopia. This could be because treatment-induced visual plasticity acts on common mechanisms for different viewing distances, ensuring a broad functional transfer of the improvements achieved.

However, several limitations of this meta-analysis should be acknowledged. First, although only randomized controlled trials were included, variability in study design, inclusion criteria, treatment duration, and outcome measures introduced heterogeneity, particularly in stereopsis and adherence analyses. Inconsistent definitions of amblyopia subtypes and variations in baseline severity may have influenced treatment effects and reduced comparability across studies, thereby limiting the precision of pooled estimates for some outcomes. Second, follow-up periods were generally short, limiting the ability to assess long-term stability of visual gains and the potential for recurrence, which reduces confidence in the durability of treatment effects. Third, adherence reporting was heterogeneous and often relied on parental or self-reported measures, which are prone to bias; objective monitoring was rarely implemented, potentially leading to overestimation of treatment exposure and efficacy. Fourth, some subgroup analyses were underpowered due to the limited number of trials, especially for emerging interventions such as fluoxetine or syntonic phototherapy, increasing uncertainty and susceptibility to small-study effects. Finally, publication bias cannot be entirely excluded, particularly in smaller studies of novel digital and adjuvant therapies. Although no language restrictions were applied, gray literature and trial registries were not systematically searched, which may have contributed to selective availability of positive findings.

This study also has notable strengths. It is, to our knowledge, the most comprehensive meta-analysis to date comparing both conventional and emerging amblyopia treatments, integrating data from diverse therapeutic categories, including pharmacological penalization, digital/binocular approaches, perceptual learning, and alternative or hybrid interventions, while applying standardized outcome measures. The inclusion of recent randomized controlled trials ensures that the analysis reflects current clinical practice trends, and the use of sensitivity analyses, GRADE certainty ratings, and risk of bias assessments adds methodological rigor. By evaluating multiple interventions in a unified framework, this work provides a broader clinical perspective than previous reviews that typically focused on single treatment modalities.

Future research should prioritize standardized definitions of amblyopia and core outcome sets to facilitate comparability and meta-analytic synthesis. Longitudinal trials with extended follow-up are needed to determine the durability of treatment effects and identify risk factors for recurrence. Interventions should also integrate adherence-enhancing strategies, such as gamified training, personalized reminders, and educational tools, given the central role of compliance in treatment success. Further studies on the combination of treatments, particularly digital therapies with pharmacological or optical penalization, could explore potential synergistic effects. Additionally, future research should examine the impact of treatments on functional outcomes beyond visual acuity, such as quality of life, reading performance, and visuomotor integration, to better reflect patient-relevant benefits. Cost-effectiveness analyses will also be essential to guide resource allocation, especially in healthcare systems with limited capacity for intensive follow-up.

## 5. Conclusions

This meta-analysis demonstrates that atropine and occlusion have equivalent efficacy in improving visual acuity in amblyopia, while digital and binocular therapies provide statistically detectable but clinically modest additional benefits and should be considered complementary rather than substitutive approaches. Pharmacological adjuvants and hybrid approaches, including filters or near activities, may further enhance outcomes, although evidence remains limited for some interventions. Flexible occlusion regimens can maintain efficacy while improving acceptability. Across all modalities, adherence emerges as the primary determinant of success across all treatments. Collectively, these findings support a tailored, multimodal approach to amblyopia management, integrating strategies that optimize patient engagement, adapt to individual needs, and extend evaluation criteria beyond visual acuity to include functional vision outcomes.

Nevertheless, variability in the definition and reporting of refractive adaptation across trials should be considered when interpreting treatment effects, as studies lacking a clearly defined adaptation period may partially reflect improvements attributable to optical correction rather than to the intervention itself.

## Figures and Tables

**Table 1:** Electronic search strategy.

Database	Search String (Summary)	Filters	Last Search
PubMed	(“amblyopia” OR “lazy eye”) AND (patching OR occlusion OR atropine OR binocular OR dichoptic OR virtual reality OR perceptual learning OR penalization OR Luminopia) AND (versus OR comparison OR head-to-head)	None	5 August 2025
Web of Science	TS = (amblyopia OR “lazy eye”) AND TS = (patching OR occlusion OR atropine OR binocular OR dichoptic OR virtual reality OR perceptual learning OR penalization OR Luminopia) AND TS = (versus OR comparison)	None	5 August 2025
Scopus	TITLE-ABS-KEY (amblyopia OR “lazy eye”) AND TITLE-ABS-KEY (patching OR occlusion OR atropine OR binocular OR dichoptic OR virtual reality OR perceptual learning OR penalization OR Luminopia) AND TITLE-ABS-KEY (versus OR comparison)	None	5 August 2025

**Table 2:** GRADE assessment of the quality of the evidence and the strength of the recommendations.

Certainty Assessment							No. of Patients		Effect		Certainty	Importance
No. of Studies	Study Design	Risk of Bias	Inconsistency	Indirectness	Imprecision	Other Considerations	[Intervention]	[Comparison]	Relative (95% CI)	Absolute (95% CI)		
**Dichoptic/binocular/VR/digital therapy vs. Patching**												
19	randomized trials	not serious	serious ^a^	not serious	not serious	none	675/1374 (49.1%)	699/1374 (50.9%)	**MD 0.02** (0.00 to 0.04)	**-- per 1000** (from -- to --)	⨁⨁⨁◯ Moderate ^a^	CRITICAL
**Atropine vs. Patching**												
2	randomized trials	not serious	serious	not serious	not serious	none	224/461 (48.6%)	237/461 (51.4%)	**MD 0.04** (−0.04 to 0.12)	**-- per 1000** (from -- to --)	⨁⨁⨁◯ Moderate	CRITICAL
**Pharmacological Adjuvant (Levodopa, CDP-choline, etc.) + Patching vs. Patching**												
8	randomized trials	not serious	serious ^a^	not serious	not serious	none	228/450 (50.7%)	222/450 (49.3%)	**MD 0.08** (0.03 to 0.13)	**-- per 1000** (from -- to --)	⨁⨁⨁◯ Moderate ^a^	CRITICAL
**Perceptual Learning vs. Patching**												
2	randomized trials	not serious	serious ^a^	not serious	serious ^b^	none	106/165 (64.2%)	59/165 (35.8%)	**MD 0.07** (−0.06 to 0.19)	**-- per 1000** (from -- to --)	⨁⨁◯◯ Low ^a,b^	CRITICAL
**Fluoxetine + Patching vs. Placebo + Patching**												
2	randomized trials	not serious	serious ^a^	not serious	serious ^c^	none	42/77 (54.5%)	35/77 (45.5%)	**MD 0.05** (−0.09 to 0.19)	**-- per 1000** (from -- to --)	⨁⨁◯◯ Low ^a,c^	CRITICAL
**Patching + near activities vs. Patching + non-near activities**												
2	randomized trials	not serious	not serious ^a^	not serious	serious ^d^	none	47/93 (50.5%)	46/93 (49.5%)	**MD 0.12** (0.05 to 0.18)	**-- per 1000** (from -- to --)	⨁⨁⨁◯ Moderate ^a,d^	CRITICAL

**CI**: confidence interval; **MD**: mean difference. ^a^ High statistical and clinical heterogeneity was present across included studies (I^2^ frequently ≥ 50%), reflecting variability in populations, interventions, and study designs. ^b^ Evidence was based on a small number of trials with limited sample sizes and wide confidence intervals crossing the line of no effect, leading to uncertainty in the pooled estimate. ^c^ Only two small trials were available, with wide confidence intervals crossing the null effect, resulting in substantial uncertainty regarding the true effect. ^d^ The evidence was derived from a small number of studies with limited sample sizes, resulting in wide confidence intervals despite consistent direction of effect.

## Data Availability

The original contributions presented in this study are included in the article/Supplementary Materials. Further inquiries can be directed to the corresponding author.

## Supplementary Materials

The following supporting information can be downloaded at https://www.mdpi.com/article/10.3390/life16020222/s1, File S1: PRISMA 2020 checklist; File S2: Full electronic database search strategies (PubMed, Web of Science, Scopus); File S3: Study-level risk of bias assessment; File S4: Risk of bias judgment; File S5: Reasons for full-text exclusion; File S6: Baseline characteristics of the 66 included studies.
