# Supplementary material for: Systematic Review and Meta-Analysis of RCTs on Efficacy of Conventional vs. Emerging Treatments for Amblyopia
Source: Life (Basel). 2026 Jan 28;16(2):222. doi: 10.3390/life16020222 (PMC12942344; doi:10.3390/life16020222)
Supplement: Supplementary file 1 [file life-16-00222-s001.zip › Additional file S2.pdf]

Additional File S2. Full electronic search strategies

Pubmed

Search: **(amblyopia OR "lazy eye") AND (patching OR occlusion OR "occlusion therapy" OR "occlusion treatment" OR atropine OR "Bangerter filter" OR binocular OR "binocular therapy" OR "binocular training" OR dichoptic OR "dichoptic training" OR "virtual reality" OR "video game" OR "digital game" OR "perceptual learning" OR "optical correction" OR "spectacle correction" OR "refractive correction" OR penalization OR suresight OR luminopia) AND (versus OR vs OR comparative OR comparison OR "head to head" OR compared)** Sort by: **Publication Date**

("amblyopia"[MeSH Terms] OR "amblyopia"[All Fields] OR "amblyopias"[All Fields] OR "lazy eye"[All Fields]) AND ("patch"[All Fields] OR "patch s"[All Fields] OR "patche"[All Fields] OR "patches"[All Fields] OR "patching"[All Fields] OR "patchs"[All Fields] OR ("dental occlusion"[MeSH Terms] OR ("dental"[All Fields] AND "occlusion"[All Fields]) OR "dental occlusion"[All Fields] OR "occlusion"[All Fields] OR "occlused"[All Fields] OR "occlusions"[All Fields] OR "occlusive"[All Fields] OR "occlusives"[All Fields]) OR "occlusion therapy"[All Fields] OR "occlusion treatment"[All Fields] OR ("atropine"[Supplementary Concept] OR "atropine"[All Fields] OR "atropin"[All Fields] OR "atropine"[MeSH Terms] OR "atropinization"[All Fields] OR "atropinized"[All Fields] OR "hyoscyamine"[Supplementary Concept] OR "hyoscyamine"[All Fields] OR "hyoscyamine"[MeSH Terms]) OR "Bangerter filter"[All Fields] OR ("binocularity"[All Fields] OR "telescopes"[MeSH Terms] OR "telescopes"[All Fields] OR "binocular"[All Fields] OR "binoculars"[All Fields]) OR "binocular therapy"[All Fields] OR "binocular training"[All Fields] OR ("dichoptic"[All Fields] OR "dichoptically"[All Fields]) OR "dichoptic training"[All Fields] OR "virtual reality"[All Fields] OR "video game"[All Fields] OR "digital game"[All Fields] OR "perceptual learning"[All Fields] OR "optical correction"[All Fields] OR "spectacle correction"[All Fields] OR "refractive correction"[All Fields] OR ("penalisation"[All Fields] OR "penalization"[All Fields] OR "penalizations"[All Fields] OR "penalize"[All Fields] OR "penalized"[All Fields] OR "penalizes"[All Fields] OR "penalizing"[All Fields]) OR "suresight"[All Fields] OR "luminopia"[All Fields]) AND ("versu"[All Fields] OR "versus"[All Fields] OR "vs"[All Fields] OR ("comparabilities"[All Fields] OR "comparability"[All Fields] OR "comparable"[All Fields] OR "comparables"[All Fields] OR "comparably"[All Fields] OR "comparative"[All Fields] OR "comparatively"[All Fields] OR "comparatives"[All Fields] OR "comparator"[All Fields] OR "comparators"[All Fields] OR "compared"[All Fields] OR "compares"[All Fields] OR "comparing"[All Fields]) OR ("comparison"[All Fields] OR "comparisons"[All Fields]) OR "head to head"[All Fields] OR ("comparabilities"[All Fields] OR "comparability"[All Fields] OR "comparable"[All Fields] OR "comparables"[All Fields] OR "comparably"[All Fields] OR "comparative"[All Fields] OR "comparatively"[All Fields] OR "comparatives"[All Fields] OR "comparator"[All Fields] OR "comparators"[All Fields] OR "compared"[All Fields] OR "compares"[All Fields] OR "comparing"[All Fields]))

Translations

amblyopia: "amblyopia"[MeSH Terms] OR "amblyopia"[All Fields] OR "amblyopias"[All Fields]

patching: "patch"[All Fields] OR "patch's"[All Fields] OR "patche"[All Fields] OR "patches"[All Fields] OR "patching"[All Fields] OR "patchs"[All Fields]

occlusion: "dental occlusion"[MeSH Terms] OR ("dental"[All Fields] AND "occlusion"[All Fields]) OR "dental occlusion"[All Fields] OR "occlusion"[All Fields] OR "occlused"[All Fields] OR "occlusions"[All Fields] OR "occlusive"[All Fields] OR "occlusives"[All Fields]

atropine: "atropine"[Supplementary Concept] OR "atropine"[All Fields] OR "atropin"[All Fields] OR "atropine"[MeSH Terms] OR "atropinization"[All Fields] OR "atropinized"[All Fields] OR "hyoscyamine"[Supplementary Concept] OR "hyoscyamine"[All Fields] OR "hyoscyamine"[MeSH Terms]

binocular: "binocularity"[All Fields] OR "telescopes"[MeSH Terms] OR "telescopes"[All Fields] OR "binocular"[All Fields] OR "binoculars"[All Fields]

dichoptic: "dichoptic"[All Fields] OR "dichoptically"[All Fields]

penalization: "penalisation"[All Fields] OR "penalization"[All Fields] OR "penalizations"[All Fields] OR "penalize"[All Fields] OR "penalized"[All Fields] OR "penalizes"[All Fields] OR "penalizing"[All Fields]

versus: "versu"[All Fields] OR "versus"[All Fields]

comparative: "comparabilities"[All Fields] OR "comparability"[All Fields] OR "comparable"[All Fields] OR "comparables"[All Fields] OR "comparably"[All Fields] OR "comparative"[All Fields] OR "comparatively"[All Fields] OR "comparatives"[All Fields] OR "comparator"[All Fields] OR "comparators"[All Fields] OR "compared"[All Fields] OR "compares"[All Fields] OR "comparing"[All Fields]

comparison: "comparison"[All Fields] OR "comparisons"[All Fields]

compared: "comparabilities"[All Fields] OR "comparability"[All Fields] OR "comparable"[All Fields] OR "comparables"[All Fields] OR "comparably"[All Fields] OR "comparative"[All Fields] OR "comparatively"[All Fields] OR "comparatives"[All Fields] OR "comparator"[All Fields] OR "comparators"[All Fields] OR "compared"[All Fields] OR "compares"[All Fields] OR "comparing"[All Fields]

<u>Web of Science</u>

TS=(amblyopia OR "lazy eye") AND TS=(patching OR occlusion OR "occlusion therapy" OR "occlusion treatment" OR atropine OR "Bangerter filter" OR binocular OR "binocular therapy" OR "binocular training" OR dichoptic OR "dichoptic training" OR "virtual reality" OR "video game" OR "digital game" OR "perceptual learning" OR "optical correction" OR "spectacle correction" OR "refractive correction" OR penalization OR suresight OR luminopia) AND TS=(versus OR vs OR comparative OR comparison OR "head to head" OR compared)

<u>Scopus</u>

TITLE-ABS-KEY(amblyopia OR "lazy eye") AND TITLE-ABS-KEY(patching OR occlusion OR "occlusion therapy" OR "occlusion treatment" OR atropine OR "Bangerter filter" OR binocular OR "binocular therapy" OR "binocular training" OR dichoptic OR "dichoptic training" OR "virtual reality" OR "video game" OR "digital game" OR "perceptual learning" OR "optical correction" OR "spectacle correction" OR "refractive correction" OR penalization OR suresight OR luminopia) AND TITLE-ABS-KEY(versus OR vs OR comparative OR comparison OR "head to head" OR compared)
