# Supplementary material for: Systematic Review and Meta-Analysis of RCTs on Efficacy of Conventional vs. Emerging Treatments for Amblyopia
Source: Life (Basel). 2026 Jan 28;16(2):222. doi: 10.3390/life16020222 (PMC12942344; doi:10.3390/life16020222)
Supplement: Supplementary file 1 [file life-16-00222-s001.zip › Additional file S3.pdf]

Additional file S3. Study-level risk of bias assessment

Study (Author, Year)	Random sequence generation	Allocation concealment	Blinding of participants & personnel (performance bias)	Blinding of outcome assessment (detection bias)	Incomplete outcome data (attrition bias)	Selective reporting	Other bias
Agervi et al. (2013)_Bangerter	Low	Unclear	Unclear	Low	Low	Low	Low
Agervi et al. (2013)_S + ADP	Low	Unclear	Unclear	Low	Low	Low	Low
Bhartiya et al. (2002)	Low	Low	Low	Low	Low	Low	Low
Chen et al. (2008)	High	High	High	Low	High	Low	Low
Chen et al. (2025)	Low	Unclear	High	Low	Low	Low	Low
Dadeva & Dangda (2016)	Low	Unclear	Unclear	Low	Low	Low	Low
Dahlmann-Noor et al. (2024)	Low	High	High	Low	Low	Low	High
Elhusseiny et al. (2021)	Low	Unclear	Low	Low	Low	Low	High
Evans et al. (2011)	Low	High	Low	Low	Low	Low	High
Foley-Nolan et al. (1997)	Unclear	Low	High	Low	Low	Low	Low
Fresina et al. (2007)	High	High	High	Low	Low	Low	High
Gao et al. (2018)	Low	Low	Low	Low	Low	Low	Low
Garcia-Romo et al. (2018)	Low	Unclear	High	Low	Low	Low	High
Herbison et al. (2016)	Low	Unclear	Low	Low	Low	Low	Low
Hernández-Andrés et al. (2025)	Low	Unclear	High	Unclear	Low	Low	Unclear
Holmes et al. (2016)	Low	Low	High	Low	Low	Low	Low
Holmes et al. (2019)	Low	Low	High	Low	Low	Low	Low
Huang et al. (2022)	Low	Unclear	High	Low	Low	Low	Unclear
Huttunen et al. (2018)	Low	Low	Low	Low	Low	Low	Low
Jost et al. (2022)	Low	Low	High	Low	Low	Low	Low
Jost et al. (2024)	Low	Low	High	High	Low	Low	Low
Kadhum et al. (2024)	Low	Low	High	Unclear	High	Low	Unclear
Kelly et al. (2016)	Low	Low	High	Unclear	Low	Low	Low
Khorrami-Nejad et al. (2024)	Low	Unclear	High	Low	Low	Low	Low
Lagas et al. (2019)	Low	Low	Low	Low	Low	Low	Low
Leguire et al. (1993)	Low	Low	Low	Low	Low	Low	Low
Leguire et al. (1998)	Unclear	Unclear	Unclear	Low	Low	Low	Unclear
Lin & Cai (2025)	Low	Unclear	Unclear	Low	Low	Low	Low
Ma et al. (2024)	Low	High	High	Low	Low	Low	Low
Manh et al. (2018)	Low	Low	High	Low	Low	Low	Low
Manny et al. (2022)	Low	Low	High	Low	Low	Low	Low
Menon et al. (2008)	Low	Unclear	High	Unclear	Low	Low	Unclear
Meqdad et al. (2024)	Low	Unclear	High	Low	Low	Low	Low
Min et al. (2021)	Low	Unclear	High	Low	Low	Low	Low
Mirmohammadsadeghi et al. (2024)	Low	Low	Low	Low	Unclear	Low	Unclear
Mohamed et al. (2025)	Unclear	Unclear	High	High	Low	Low	High
Pang et al. (2021)	Low	Unclear	Low	High	Low	Low	Unclear
Pawar et al. (2014)	Low	Unclear	High	High	High	Low	Unclear

Pediatric Eye Disease Investigator Group (2003)	Low	Low	Low	Low	Low	Low	Low
Pediatric Eye Disease Investigator Group (2005)	Low	Low	Low	Unclear	Low	Low	Low
Pediatric Eye Disease Investigator Group (2008)	Low	Low	High	Low	Low	Low	Low
Pediatric Eye Disease Investigator Group (2009)	Low	Low	High	Low	Low	Low	Low
Pediatric Eye Disease Investigator Group (2010)	Low	Low	Low	Low	Low	Low	Low
Pediatric Eye Disease Investigator Group (2013)	Low	Low	Low	Low	Low	Low	Low
Pediatric Eye Disease Investigator Group (2015)	Low	Low	Low	Low	Low	Low	Low
Polat et al. (2004)	Low	Unclear	Unclear	Low	Low	Low	High
Poltavski et al. (2025)	Low	Low	High	Low	Low	Low	Low
Proudlock et al. (2024)	Low	Low	High	High	Unclear	Low	Low
Repka et al. (2007)	Low	Low	High	Low	High	Low	Low
Roy et al. (2023)	Low	Unclear	High	Unclear	Low	Low	Low
Sharif et al. (2019)	Low	Low	High	Low	Low	Low	Low
Singh et al. (2017)	Unclear	Unclear	High	Low	Low	Low	Low
Stanković & Milenković (2007)	Low	Unclear	High	Unclear	Low	Low	Low
Stewart et al. (2007)	Low	Low	High	Low	Low	Low	Low
Tejedor & Gutiérrez-Carmona (2023)	Low	Low	Low	Low	Low	Low	Low
Tejedor & Ogallar (2008)	Low	Unclear	Unclear	Low	Low	Low	Unclear
Uttamapinan et al. (2024)	Low	Low	High	Low	Low	Low	Unclear
Wang et al. (2016)	Low	Low	High	Low	Low	Low	Low
Wang et al. (2021)	Low	Low	High	Low	Low	Low	Low
Wu et al. (2010)	Low	Low	High	Low	Low	Low	High
Wygnanski-Jaffe et al. (2023)	Low	Low	Unclear	Low	Low	Low	Low
Wygnanski-Jaffe et al. (2025)	Low	Low	High	Low	Low	Low	Low
Xiao et al. (2022)	Low	Low	High	Low	Low	Low	High
Yuan et al. (2021)	Low	Unclear	High	Unclear	Low	Low	High
Zhao et al. (2010)	Low	Unclear	High	Low	Low	Low	Low
Zhu et al. (2023)	Low	Low	High	Low	Low	Low	Unclear
