# Supplementary material for: Systematic Review and Meta-Analysis of RCTs on Efficacy of Conventional vs. Emerging Treatments for Amblyopia
Source: Life (Basel). 2026 Jan 28;16(2):222. doi: 10.3390/life16020222 (PMC12942344; doi:10.3390/life16020222)
Supplement: Supplementary file 1 [file life-16-00222-s001.zip › Additional file S4.pdf]

Additional file S4. Risk of bias judgement.

Agervi et al. (2013)_Bangerter

A) The study is described as a "randomized clinical trial". Children were randomized by "drawing a numbered allocation ticket provided by the researchers", which indicates a random process

B) Although random allocation was used, the process of drawing a numbered ticket may not have ensured concealment from enrolling investigators, and the exact method for preventing foreknowledge of group assignment is not described.

C) The study was open-label: both participants and treating clinicians were aware of the group assignment (spectacles alone vs. Bangerter filter). Masking would be challenging due to the visible presence of the filter on glasses. No attempt at blinding is reported.

D) The same investigator mainly performed VA assessments in a standardized way. Although not explicitly masked, the objective nature of the VA measurement and the use of standardized procedures reduces risk of bias in outcome assessment.

E) 65 of 80 children (81%) completed the 2-year examination. Dropout rates were similar in both groups and reasons for dropout were reported. Analysis appears to be conducted on those who completed follow-up (completers' analysis), but there is no evidence of differential attrition affecting results.

F) The primary and secondary outcomes (visual acuity change, binocular function, refractive error) were prespecified and reported. No evidence of selective reporting.

G) The study declares no financial conflicts of interest. Both groups were well-matched at baseline and standard protocols were followed. Compliance was checked by parental diary and direct questioning. No major other sources of bias identified.

Agervi et al. (2013)_S + ADP

A) The study is described as a randomized clinical trial; allocation to groups was performed by "drawing a numbered allocation ticket", indicating adequate random sequence generation.

B) There is no detailed information regarding the concealment process. Although tickets were drawn, it is not explicitly stated whether allocation was concealed from those enrolling participants.

C) Due to the nature of the intervention (different patching regimens), blinding of participants and personnel was not feasible. This could introduce performance bias, but is difficult to avoid in this context.

D) Visual acuity was assessed using standardized and objective procedures. Although it is not specified whether the examiner was masked, the objectivity of the outcome reduces risk of detection bias.

E) 95% of patients (38/40) completed the 2-year follow-up. Only two dropouts occurred, both in the daily patching group, and reasons for withdrawal were provided. Low risk of attrition bias.

F) All pre-specified primary and secondary outcomes (change in visual acuity, binocular function, refractive error) were reported. There is no evidence of selective outcome reporting.

G) No other sources of bias identified: baseline characteristics were balanced, no conflicts of interest declared, and both adherence and follow-up were adequate.

Bhartiya et al. (2002)

A) The study is described as "randomized, placebo-controlled." Although the exact randomization method is not specified, the use of coded placebo and active treatment capsules strongly suggests appropriate randomization.

B) Capsules were prepared and coded by the pharmacy; the code was revealed only after completion of statistical analysis. This indicates proper allocation concealment.

C) Double-masked (double-blind) design: both participants and investigators were unaware of group allocation. Code was not revealed until after the study ended.
D) Blinding of outcome assessors is not explicitly stated, but given the coded treatments and blinding of both patients and staff, assessors were likely blinded.
E) No significant loss to follow-up or withdrawals reported. All randomized participants were included in the analysis
F) All main outcomes (visual acuity, contrast sensitivity, compliance, tolerance) are reported as described in the methods.
G) No other significant sources of bias identified. Baseline groups were comparable, and the study appears well designed.

<u>Chen et al. (2008)</u>
A) There is no information about concealment of allocation or whether group assignment was hidden from recruiters/participants prior to group allocation.
B) There is no information about concealment of allocation or whether group assignment was hidden from recruiters/participants prior to group allocation.
C) Due to the nature of interventions (perceptual learning vs patching), blinding of participants and therapists was not possible. Participants and providers were aware of group assignment.
D) Visual acuity and contrast sensitivity were measured by an examiner masked to patient diagnosis and previous VA readings, reducing the risk of detection bias.
E) There was substantial withdrawal/dropout: 4 of 30 in the perceptual learning group (13%) and 4 of 31 in the patching group (13%), mostly adults (withdrawal due to intolerance or boredom). Reasons are reported, but dropout could introduce bias, especially as more adults withdrew from patching.
F) All major outcomes (visual acuity, contrast sensitivity, retention) appear to be reported as per the methods section; results are detailed for both groups.
G) This is a pilot study with relatively small and uneven group sizes, and baseline mean age was slightly higher in the perceptual learning group (potential confounding). Compliance for patching was parent/teacher-reported. Data analysis was limited to one factor at a time, as admitted by the authors. No formal power calculation provided.

<u>Chen et al. (2025)</u>
A) Randomization was performed using a sequence generated by R software.
B) The study does not detail whether allocation was concealed from investigators or participants before assignment, so risk of selection bias cannot be fully excluded.
C) Due to the nature of the interventions (gamified binocular therapy vs. patching), blinding of participants and caregivers was not possible. This may introduce performance bias.
D) Outcome assessors (examiners for visual acuity and stereoacuity) were masked to group allocation, minimizing detection bias.
E) 5/42 participants withdrew, mainly due to lack of willingness to continue follow-up, not adverse events. Attrition was balanced between groups and reasons were reported. Intent-to-treat analysis appears to have been used.
F) All prespecified primary and secondary outcomes (visual acuity, stereoacuity, adherence, satisfaction) are reported as described in the methods.
G) Baseline characteristics were well matched between groups, no financial or commercial conflicts of interest declared, and the study was prospectively registered.

Dadeya & Dangda (2016)
A) The study is described as prospective and randomized; subjects were "randomly divided into two groups of 20." The method of randomization is not detailed but groups appear balanced.
B) No details provided about how allocation was concealed (e.g., sealed envelopes or central randomization), so there is potential for selection bias.
C) Due to the nature of the intervention (video games + patching vs. patching alone), neither participants nor those delivering the interventions could be blinded.
D) The manuscript does not specify whether outcome assessors (visual acuity and stereoacuity) were blinded to group assignment. Detection bias cannot be excluded.
E) The study reports 40 children started and all completed follow-up. There is no evidence of significant attrition or missing outcome data.
F) The main and secondary outcomes (distance and near BCVA, stereoacuity) are all reported, with statistical comparisons at each time point.
G) The main and secondary outcomes (distance and near BCVA, stereoacuity) are all reported, with statistical comparisons at each time point.

Dahlmann-Noor et al. (2024)
A) Randomisation was performed using a web-based system (Sealed Envelope), with minimisation and a random element. Allocation was stratified for key prognostic factors.
B) Allocation was implemented via the web-based Sealed Envelope system, ensuring concealment of upcoming assignments.
C) Masking was not possible for participants or parents due to the nature of the interventions (BBV device vs. patching/atropine).
D) Outcome assessments were performed by an orthoptist masked to group allocation. Families were asked not to reveal allocation to the masked assessor.
E) 63% retention rate: Only 20/32 participants completed the 16-week follow-up; high dropout and missing adherence data, mostly related to COVID-19 disruptions and acceptability issues.
F) All prespecified primary and secondary outcomes (including feasibility, safety, efficacy) were reported as planned in the protocol.
G) Small sample size, low statistical power (due to early study termination and COVID-19 impact); only 32 participants enrolled vs. 44 planned; possible baseline imbalances between groups.

Elhusseiny et al. (2021)
A) The trial was prospective and randomized, with allocation (1:1) to full treatment or sham-crossover groups, using randomization procedures.
B) The exact method of allocation concealment was not described (e.g., sealed envelopes, computerized randomization); thus, this domain is rated as unclear.
C) The study is described as double-masked: both participants and clinicians were masked to treatment allocation (sham vs. active).
D) Outcome assessments (visual acuity, stereoacuity) were performed by masked examiners, reducing detection bias.
E) There was a notable proportion of attrition (three participants did not complete 16-week follow-up; two dropped out after randomization), and the small sample size amplifies attrition impact.
F) All prespecified outcomes (visual acuity, stereoacuity, adverse events, adherence) were reported in the results.

G) Small sample size (n=20, with dropouts), pilot design, modest follow-up, and lack of objective confirmation of device usage (adherence could be overestimated).

Evans et al. (2011)
A) The study used random allocation to assign participants to IPS or control groups. Although the specific method is not described in detail, the context and terminology strongly suggest an adequate randomisation process was used.
B) There is no explicit description of allocation concealment (e.g., sealed envelopes, centralized randomization). However, it is stated that both participants and assessors were masked, reducing but not eliminating the risk of bias.
C) The study was double-masked: both participants and outcome assessors were unaware of group allocation. Control and experimental treatments were designed to appear similar, including similar instructions and procedures.
D) Visual acuity was measured by examiners masked to group allocation, and the method minimized the possibility of learning or bias in repeated measurements.
E) All 30 randomized participants (15 per group) completed the intervention and primary post-treatment assessment. Follow-up was available for 7 (IPS) and 10 (control) at 1–3 years, with reasons for loss to follow-up described and balanced across groups.
F) All specified outcomes (VA improvement, subgroup analyses by amblyopia type, retention at follow-up) are reported. Methods and outcomes match those described in the protocol section.
G) Groups were similar in age, amblyopia type, and prior treatments. Compliance with spectacle wear outside the clinic was not monitored. The small sample size and possible underpowering for subgroup analyses are noted.

Foley-Nolan et al. (1997)
A) Patients were allocated to treatment on a "strict alternate patient basis" (i.e., every other new patient to each group). This is quasi-random, not true randomization.
B) Appointment scheduling and allocation were handled by an independent observer (clinic sister), which likely reduced selection bias.
C) Neither participants nor treating clinicians were blinded due to the obvious nature of interventions (drops vs. patch).
D) Visual acuity was assessed by two independent observers masked to treatment allocation, reducing detection bias.
E) All 36 enrolled patients were followed; no attrition or missing data is reported.
F) All prespecified and clinically relevant outcomes (acuity, compliance, complications) are reported.
G) Compliance for patching was parent-reported, which is prone to reporting bias. Compliance for atropine was checked both by parent report and pupil check (more objective). Sample size is small.

Fresina et al. (2008)
A) Randomization is mentioned, but details about the sequence generation (e.g., method of random number generation or allocation concealment) are not described.
B) No explicit description of how allocation was concealed; open-label design increases the risk that allocation was not fully concealed from study personnel or participants.
C) This was an open-label study; neither participants nor investigators were blinded to treatment allocation, increasing the risk of performance and detection bias.

D) Outcome assessors were not blinded; visual acuity and contrast sensitivity assessments were performed in an open-label setting, raising the risk of detection bias.
E) Attrition was low (6 out of 67 participants dropped out, with reasons stated as unrelated to the intervention), and per-protocol analysis included 61 participants.
F) The primary and secondary outcomes specified in the methods are reported; there is no evidence of selective outcome reporting.
G) Possible sources of bias include use of Snellen E charts instead of standard optotypes, potential for compliance bias, and declared financial interest (one author advisor for manufacturer).

Gao et al. (2018)
A) Randomization was performed centrally through a secure website using a minimization method stratified by age group; clearly described in the protocol.
B) Allocation was implemented centrally, and staff involved in allocation were separate from those conducting outcome assessment.
C) This was a double-masked (double-blind) trial: participants and their families were not told about allocation, and both the active and placebo games were similar in appearance and operation.
D) Clinicians measuring visual outcomes were masked to group assignment at all visits, minimizing detection bias.
E) Attrition was low and similar between groups (89% and 97% completed the 6-week primary outcome visit). Intention-to-treat analyses with imputation for missing data were conducted.
F) All prespecified primary and secondary outcomes were reported in the published results, with supplementary materials and protocol available.
G) Baseline characteristics were balanced. Objective compliance data was collected via the device, and the trial was registered (ACTRN12613001004752). No evidence of other sources of bias.

Garcia-Romo et al. (2018)
A) The study states that participants were randomly assigned to intervention groups. Although the exact method is not specified, the allocation appears random.
B) The procedure for concealing allocation is not described, so it is unclear whether allocation could have been foreseen by study personnel at enrollment.
C) Blinding was not possible due to the nature of interventions (patch vs. occluding contact lens), and the authors state that masking was not performed.
D) Examiners were not blinded to intervention groups, which could influence the assessment of subjective outcomes (quality of life), although VA is objective.
E) All participants completed follow-up and there were no reported withdrawals or exclusions after randomization.
F) All primary and secondary outcomes (BCVA, mfVEP, NEI VFQ-25) appear to be reported as planned; no evidence of selective outcome reporting.
G) Small sample size (n=10), pilot design, and lack of masking may introduce other sources of bias. The authors acknowledge these limitations.

Herbison et al. (2016)
A) The randomization sequence was generated using random permuted blocks (sizes 2, 4, 6) with Stata, and stratified by center and previous amblyopia treatment.
B) The method for allocation concealment is not explicitly described. No details are provided on how the assignment was concealed from enrolling staff and participants.

C) All three arms used similar hardware and dichoptic stimulation; treatments were delivered in a way that would be difficult for participants to distinguish.
D) Visual acuity measurements were performed by an independent orthoptist masked to group allocation (assessor-blinded).
E) Analysis was performed by intention to treat; participant flow was clear; few withdrawals; missing data were assumed random; no significant attrition bias identified.
F) All prespecified primary and secondary outcomes were reported, including VA change, stereoacuity, compliance, and adverse events.
G) Most participants were residual amblyopes with previous treatment failures, and 93% had strabismic amblyopia. The total treatment time was very short (3 hours).

Hernández-Andrés et al. (2025)
A) Participants were randomly assigned to the three groups using MATLAB's *randperm* function, indicating a proper randomization process.
B) There is no information regarding whether allocation was concealed (e.g., sealed envelopes or centralized process).
C) Blinding was not possible due to the nature of the interventions (patching, therapy, computer game).
D) The examiner was aware of group assignments, but claimed to have no preconceived notions; some risk of detection bias remains.
E) No significant loss to follow-up was reported; outcome data were available for all randomized participants.
F) All pre-specified outcomes (visual acuity, stereoacuity, interocular difference) are reported in full.
G) Adherence was mostly self-reported by parents and not objectively monitored. Supervision differed between groups (more frequent clinic visits in one group). Sample size was moderate.

Holmes et al. (2016)
A) Central randomization was conducted using a computer-generated permuted block design, stratified by age group and site, minimizing risk of selection bias.
B) Randomization was performed centrally via the PEDIG website; allocation was concealed from site investigators at the time of enrollment.
C) Participants, families, and treating clinicians could not be blinded due to the nature of the interventions (binocular iPad game vs. patching), introducing potential performance and reporting bias, particularly for subjective outcomes and compliance. However, the main outcome (VA) is objective.
D) Examiners assessing the primary outcome (visual acuity) were masked to treatment allocation for 99% of follow-up visits, greatly reducing detection bias for the main outcome.
E) Follow-up at the primary outcome (16 weeks) was completed by 96% of both groups. Missing data were minimal and balanced, with intention-to-treat analysis applied.
F) Study protocol was prospectively registered (ClinicalTrials.gov NCT02200211), and all pre-specified primary and secondary outcomes were reported.
G) Compliance with the binocular game was objectively monitored (iPad logs), but compliance with patching relied on parental reporting, which is susceptible to overestimation. Additionally, compliance in the binocular group was notably poor (only 22% completed >75% of prescribed treatment), which could bias the effect estimate. No use of occlusion dose monitors in the patching group. Also, the trial used different daily doses for the two arms (2h patching vs 1h game), which might affect comparability.

Holmes et al. (2019)
A) Participants were randomized centrally via the PEDIG website, using a permuted block design stratified by baseline VA.
B) Central allocation through PEDIG website ensured allocation concealment; assignment could not be predicted by investigators.
C) Due to the nature of interventions (binocular iPad game vs spectacle correction), blinding of participants and treating clinicians was not feasible.
D) Visual acuity and stereoacuity testing were performed by examiners masked to treatment allocation at all visits, minimizing detection bias.
E) Follow-up rates were high (≥97% at all outcome points) with similar attrition across groups and no evidence of differential dropout.
F) The trial was prospectively registered (NCT02983552) and all pre-specified primary and secondary outcomes were reported as planned.
G) Adherence was objectively monitored (iPad logs) and reported; adherence was moderate but analyzed appropriately. No evidence of other biases (funding, design, etc).

Huang et al. (2022)
A) The children in the clinical studies were described as being "randomly assigned" to groups; randomization appears adequate, though details of the method are not specified
B) There is no explicit mention of allocation concealment procedures (e.g., sealed envelopes, third-party assignment), so the risk here is unclear.
C) The interventions (asynchronous 3D movie viewing + patching vs. patching alone or synchronous movies) are difficult to blind for both participants and personnel.
D) Visual acuity was measured by examiners ("highly experienced medical staff") who were "semi-blinded": aware of study participation, but not of treatment group allocation.
E) Drop-out rates were very low and reported transparently (e.g., only 2 of 40 children dropped out in Study 2, with reasons provided).
F) All primary and secondary outcomes (visual acuity, interocular difference, follow-up) are reported; data is available in text and supplementary materials.
G) Reliance on parental report for compliance with patching, small sample size in some studies (e.g., n=8 in Study 1), and possible practice effects due to repeated testing.

Huttunen et al. (2018)
A) Randomized, 1:1 allocation in blocks of 4, stratified by site and amblyopia severity, by independent statistician; randomization process clearly described.
B) Medication was pre-packed, serially numbered, and dispensed according to randomization list by personnel blinded to group assignment.
C) Double-blind: Both participants and personnel were blinded to treatment (fluoxetine vs. placebo); perceptual training was identical in both groups.
D) All vision testers and assessors were masked to group assignment; standardized procedures and charts used.
E) 42 randomized, 37 completed treatment; intention-to-treat analysis used with LOCF imputation for dropouts. Reasons for withdrawal balanced and reported.
F) All prespecified primary and secondary outcomes (visual acuity, binocularity, contrast sensitivity, crowded near VA, safety) reported as per protocol.
G) Groups balanced at baseline; compliance monitored objectively (>85% training compliance), adverse events documented, no relevant conflicts of interest, multi-center design.

Jost et al. (2022)
A) Randomization performed online via www.randomization.org, block randomization (block size 12), assignment concealed until after enrollment.
B) Allocation assignments were sealed in numbered envelopes, only accessed after subject enrollment.
C) Blinding not feasible: families, children, and clinical staff were aware of group assignment (dichoptic movies vs patching). No placebo control.
D) Visual acuity and stereoacuity testing protocols were standardized; however, as children/parents were not blinded, there is potential for detection bias, especially for subjective outcomes.
E) 65 randomized, 60 completed primary endpoint; attrition was low and balanced across groups. Modified intention-to-treat analysis (only those who completed 2-week endpoint included).
F) All primary and secondary outcomes (visual acuity, stereoacuity, suppression, adherence) reported as prespecified in protocol (registered on ClinicalTrials.gov).
G) Baseline characteristics well balanced; adaptation to spectacles prior to randomization; high adherence to protocol in both arms; analysis appropriate.

Jost et al. (2024)
A) Randomization was performed using a coded distribution in blocks of 4-8 allocations, ensuring an unpredictable and adequate random sequence. This minimizes selection bias.
B) Treatment assignments were sealed in numbered envelopes, which were only opened after confirming eligibility and obtaining informed consent. This process prevents manipulation or prediction of allocation.
C) Due to the nature of the intervention (dichoptic cartoons vs. patching), it was not possible to blind participants or personnel. This introduces a potential risk of performance bias.
D) The study explicitly states that masking of examiners was not possible due to it being a pilot study. Although primary outcomes (visual acuity) are objective, the lack of blinding of outcome assessors is considered a source of potential bias.
E) 32 out of 34 participants (94%) completed the primary outcome visit at 2 weeks. The reasons for missing data (dropout and illness) were reported and do not appear related to outcomes or group assignment. The rate and handling of missing data are acceptable.
F) All pre-specified outcomes registered in the protocol and ClinicalTrials.gov (NCT04086524) were reported in the article. There is no evidence of selective outcome reporting.
G) No other significant sources of bias were identified. Although the sample size is small (pilot study), this is clearly stated and justified. Adherence was high and monitored via parental logs and streaming logs for the dichoptic cartoon group.

Kadhum et al. (2024)
A) Randomization was performed using statistical software (R randomize package, permuted blocks), as clearly described in the methods section
B) Centralized and computerized randomization was used; while the exact allocation mechanism is not exhaustively detailed, assignment was after recruitment.
C) Due to the nature of the interventions (dichoptic gaming vs. occlusion), neither participants nor personnel could be blinded to group allocation.
D) The main orthoptist was initially masked to randomization, but in practice, blinding could not always be guaranteed. Some outcomes were objective (VA), others not

E) There was a substantial dropout rate, especially in the gaming group (56%). Both intention-to-treat and per-protocol analyses were performed, but final numbers were small.
F) All pre-specified primary and secondary outcomes (VA, efficiency, compliance, stereoacuity, contrast sensitivity) were reported. No evidence of selective reporting.
G) Possible Hawthorne effect in compliance monitoring, small final sample size (especially in gaming), possible age imbalance between groups.

<u>Kelly et al. (2016)</u>
A) Randomization was performed by a statistician using computer-generated permuted blocks and separate schedules for children with/without prior amblyopia treatment.
B) Allocation was concealed using individual sealed, sequentially numbered envelopes, opened only after consent and enrollment.
C) Due to the nature of the interventions (binocular iPad game vs. patching), participants and study personnel could not be blinded, introducing risk of performance bias.
D) The manuscript does not explicitly state whether outcome assessors were blinded to group allocation. Vision assessments were protocolized, but potential detection bias cannot be fully excluded.
E) Outcome data were available for all but one participant at the 2-week visit, and all 28 completed the 4-week visit. No dropouts; reasons for missing data were reported and unrelated to the study.
F) The study was pre-registered (clinicaltrials.gov NCT02365090) and all pre-specified outcomes were reported
G) Groups were balanced at baseline, compliance was high, and the intent-to-treat principle was followed.

<u>Khorrami-Nejad et al. (2024)</u>
A) The study states that 110 children were *randomly divided* into two groups (CAM vs. occlusion). Randomisation is explicitly mentioned.
B) The method for concealing group allocation is not described in the abstract. It is unclear if allocation was concealed from enrolling clinicians/participants
C) Due to the nature of interventions (CAM training vs. occlusion patching), masking of participants, parents, and therapists is not feasible
D) The article does not mention whether the examiners assessing visual acuity were masked to group assignment.
E) Results are reported for the full sample (n=110).
F) All planned primary and key subgroup outcomes (by type/severity) and time points are reported in the abstract.
G) No apparent other bias (groups were similar at baseline, adequate sample size for an amblyopia trial, analysis by intention to treat likely).

<u>Lagas et al. (2019)</u>
A) The study used computer-generated randomization for allocation order (active or placebo first), which was adequately described.
B) Allocation was concealed: only the pharmacist was unblinded to group assignment; all investigators and participants were masked.
C) Double-blind: both participants and researchers were masked to allocation (active or placebo tablet). Tablets were indistinguishable.
D) Outcome assessors were masked to treatment allocation throughout, minimizing risk of detection bias for visual acuity and secondary outcomes

E) All 7 randomized participants completed both crossover arms and were analyzed as randomized. No dropouts reported after randomization.
F) The trial was preregistered (ACTRN12611000669998), and all primary and secondary outcomes described in the protocol appear to be reported.
G) The main concern is the small sample size (n=7, vs. planned n=20), increasing risk of type II error and bias due to chance. One included participant did not meet visual acuity criteria. Crossover design is appropriate, but there may be residual carryover effects despite a 2-week washout. Self-reported adherence was ~70%. Analysis appears appropriate, but the trial is underpowered and not all protocol eligibility was strictly maintained.

Leguire et al. (1993)
A) Explicitly stated as "randomized", assignment to three groups was randomized by pharmacy personnel.
B) Capsules were filled and randomized by pharmacy staff; both investigators and participants were masked.
C) Double-masked (double-blind); both staff and participants were unaware of group allocation.
D) Visual acuity measured by study staff masked to allocation; different ETDRS charts used for each test to prevent learning bias.
E) 20 participants enrolled, 2 excluded before randomization for inability to complete testing; no differential dropout reported.
F) All specified outcomes (visual acuity improvement, tolerance, side effects) reported in detail.
G) Baseline characteristics similar across groups; study methods described and ethical approval obtained

Leguire et al. (1998)
A) Randomization was performed by drawing numbers from a hat, which provides some random allocation but is not as robust or concealed as computer-generated sequences
B) No mention of allocation concealment; allocation likely not concealed from study staff at assignment.
C) Participants were not blinded to group allocation (patching vs no patching), but were instructed not to reveal group allocation to the vision teste
D) Vision testers (outcome assessors) were masked to occlusion status, reducing detection bias.
E) All 13 subjects completed all test sessions; compliance and follow-up were excellent.
F) All planned outcomes (visual acuity, contrast sensitivity, fusion, adverse events) are reported as per protocol.
G) Small sample size increases risk of random error; occlusion compliance was self-reported; capsule compliance was measured by pill count; side effects were mild and similar between groups.

Lin & Cai (2025)
A) The study states that 148 patients were “randomly divided” into the two groups (ICL vs ICL+rTMS). The method of randomization is not described in detail, but the use of a random allocation is stated.
B) There is no description of how allocation was concealed (e.g., sealed envelopes, centralized randomization). It is not possible to determine whether those enrolling participants could foresee assignments.
C) The text notes that “all group procedures were conducted by independent assessors who maintained blinding to reduce assessment bias.” However, it is not explicitly clear whether

participants and all care providers were blinded. Because rTMS is an active procedure, and there is no mention of a sham rTMS or similar, blinding of participants may not have been feasible.
D) Visual outcome assessments and imaging analyses were conducted by "independent assessors who maintained blinding," suggesting that outcome assessors were masked to group allocation.
E) There is no mention of dropouts or missing data. The baseline and follow-up numbers appear to be complete (74 in each group at baseline and follow-up), suggesting minimal attrition.
F) All stated outcomes (BCVA, stereopsis, fMRI metrics) are reported in the results. There is no evidence of selective non-reporting.
G) Authors declare no conflicts of interest, no external funding, and no commercial involvement. There is no obvious other source of bias. Groups were similar at baseline.

<u>Ma et al. (2024)</u>
A) Randomization was performed using SPSS to generate random numbers according to enrollment order. The method is appropriate and clearly described.
B) There is no information provided about how allocation was concealed (e.g., use of sealed envelopes or centralized assignment). It is unclear whether the allocation could have been predicted or manipulated.
C) No mention is made of blinding participants or personnel. Given the nature of acupuncture versus conventional treatment, it is very unlikely that participants or providers were blinded. This could introduce performance and placebo bias, which is particularly relevant in acupuncture studies.
D) It is not stated whether the assessors measuring BCVA or analyzing fMRI data were blinded to group allocation. While BCVA is an objective outcome, unblinded assessment could still introduce bias.
E) Drop-outs are reported (2 children dropped out due to intolerance to acupuncture), with final group numbers provided (n=38 vs. n=36). The reason for loss is given and not likely related to outcomes. No evidence of unbalanced or high attrition.
F) All predefined outcomes (BCVA, response rate, fMRI parameters) are reported in the results. No evidence of selective outcome reporting.
G) No conflicts of interest reported; funders had no role in study design or reporting.Baseline characteristics are balanced between groups. Study is ethically approved and registered.

<u>Manh et al. (2018)</u>
A) Participants were randomized via the PEDIG website using a permuted block design stratified by baseline visual acuity and site.
B) Centralized randomization was used, with allocation performed via the PEDIG website, minimizing risk of selection bias.
C) Due to the nature of the interventions (binocular game vs patching), participants and clinical personnel could not be blinded to group assignment.
D) Visual acuity and stereoacuity testers were masked to treatment assignment at 97–100% of outcome visits, reducing risk of detection bias.
E) Completion rates were high (98% in binocular, 97% in patching group); reasons for missing data were reported and balanced across groups.
F) The study was pre-registered (NCT02200211), and all pre-specified outcomes appear to have been reported.
G) There was substantial poor adherence in the binocular group (only 13% completed >75% prescribed treatment), potentially impacting effect estimates; the study was stopped early for futility, which may introduce bias or reduce power.

Manny et al. (2022)

A) Participants were randomized centrally via the PEDIG website, using a permuted block design stratified by baseline visual acuity.

B) Central allocation and stratification by the PEDIG system ensured allocation concealment; treatment assignment could not be predicted by investigators.

C) Due to the intervention type (binocular iPad game vs. spectacle correction), blinding of participants and treating clinicians was not feasible.

D) Visual acuity and stereoacuity were assessed by masked examiners at both main outcome visits (4 and 8 weeks), minimizing detection bias.

E) High follow-up rates (>92% at all outcome visits) with minimal and balanced attrition between groups.

F) The trial was prospectively registered (NCT02983552) and all prespecified primary and secondary outcomes were reported as planned.

G) Adherence was objectively monitored (iPad log files) and reported. No major sources of other bias identified; the study was publicly funded, and data analysis was independent.

Meqdad et al. (2024)

A) Simple randomization stated. 1:1 allocation. No evidence of non-random sequence; sample size calculations and pre-registration described.

B) No explicit details on concealment (e.g., sealed envelopes or centralized randomization).

C) No blinding: participants knew their assignment (VR therapy vs patching), and interventions were inherently different/visible.

D) BCVA and stereoacuity measurements taken by an investigator masked to study design and group assignment.

E) No mention of differential attrition. ITT analysis performed. Drop-out rates not excessive; sensitivity analyses for missing data performed.

F) All outcomes defined in the protocol and methods (visual acuity, stereopsis, compliance, adverse events) are reported

G) Groups balanced at baseline. Protocol pre-registered (NCT04302701). No evidence of other sources of bias.

Menon et al. (2008)

A) The study is described as prospective and randomized, with stratified randomization used to allocate patients to groups and subgroups.

B) There is no explicit description of how allocation was concealed from investigators enrolling participants; thus, risk is unclear.

C) Due to the nature of the interventions (patching vs. atropine), neither participants nor clinical staff could be blinded to group assignment.

D) No explicit statement regarding whether outcome assessors were masked. Visual acuity testing could be subject to detection bias if not masked

E) Attrition was low (6/63, 9%), evenly distributed between groups. Dropouts were considered treatment failures, minimizing attrition bias.

F) All prespecified outcomes (distance/near visual acuity, contrast sensitivity, stereoacuity, adverse effects, compliance) were reported in the results.

G) Sample size calculation was not performed; the final statistical power was very low (6%), indicating a high risk of Type II error. Compliance was assessed subjectively (not objectively monitored).

Min et al. (2021)

A) Randomization was performed using a random number generator.

B) The method of allocation concealment is not described, so potential selection bias cannot be fully excluded.

C) Blinding of participants and caregivers was not feasible due to the nature of the interventions (visible patch vs. electronic glasses). This introduces risk of performance bias.

D) The study does not specify whether outcome assessors were masked to treatment allocation, so risk of detection bias cannot be excluded.

E) One participant was excluded after starting the trial for protocol deviation (received another treatment). Dropouts and missing data are accounted for, and analysis appears to be by protocol.

F) All prespecified primary and secondary outcomes (BCVA, contrast sensitivity, stereopsis, fusional vergence) are reported as described in the methods section.

G) Baseline characteristics were well balanced; compliance was objectively tracked in the Flicker Glass group and via diaries in the patching group. Funding sources were disclosed and do not suggest bias.

Mirmohammadsadeghi et al. (2024)

A) Randomization was performed using random block sizes (2, 4, 6) generated by a methodologist not involved in the study, ensuring adequate random sequence generation.

B) Allocation was concealed from investigators until patient enrollment, reducing selection bias.

C) Double-blind design: participants and researchers were masked to group allocation (fluoxetine vs. placebo).

D) Visual acuity was measured by a single masked optometrist; VEPs were performed by an examiner blinded to treatment allocation.

E) Five participants (1 fluoxetine, 4 placebo) discontinued the intervention. Exclusions were described, but no intention-to-treat analysis was explicitly reported; attrition is moderate given total sample.

F) All specified primary and secondary outcomes (visual acuity and VEP parameters) are reported, including details of dropouts.

G) Side effects were not formally monitored (only spontaneous reports), compliance with medication/patching was self-reported and not objectively confirmed. Sample size is modest.

Mohamed et al. (2025)

A) The study is described as "randomized," but there is no information about how the random sequence was generated (e.g., computer-generated, random numbers table, etc.).

B) No details are provided about allocation concealment procedures (e.g., sealed opaque envelopes). It is unclear if group assignment could have been predicted.

C) No blinding is described. The interventions (syntonic phototherapy vs. part-time occlusion) are very different and easily distinguishable by participants and staff.

D) There is no indication that outcome assessors were blinded. Outcome assessment (visual acuity, functional visual field, etc.) could be influenced by knowledge of group allocation.

E) All randomized participants (n=40) were analyzed. No losses to follow-up or exclusions after randomization are reported.

F) All outcomes mentioned in the methods and objectives (UCVA, BCVA, AOP, functional visual field, success rate, etc.) are reported as planned.

G) There are significant baseline differences in age between groups (mean 10.7 vs. 18.2 years, p=0.01). The syntonic group received combined intervention (phototherapy + vision therapy),

which is not controlled for in the occlusion group (potential co-intervention bias). Small sample size.

Pang et al. (2021)
A) Block randomization was employed and clearly described, minimizing selection bias.
B) The method for allocation concealment is not detailed. There is no description of how randomization was concealed from participants and investigators.
C) The study used a single-masked (participant-masked) design: participants did not know their group allocation (active vs placebo game was visually similar).
D) The clinician conducting outcome assessments was not masked to treatment group allocation, as acknowledged by the authors. This introduces a risk of detection bias.
E) All randomized participants completed follow-up; there is no mention of dropouts or missing data, and intent-to-treat analysis was used.
F) All pre-specified primary and secondary outcomes (DVA, NVA, stereoacuity, fixation stability) were reported as planned.
G) Small sample size (pilot study), more participants in the placebo group required new glasses before randomization (potential confounder), outcome stability criteria may not fully control for delayed treatment effects, and lack of double masking.

Pawar et al. (2014)
A) The study reports random allocation of patients to treatment groups, suggesting appropriate random sequence generation.
B) The method for allocation concealment is not described, so the risk of selection bias cannot be excluded.
C) The study is described as "open" and not double-blind; both participants and clinicians were aware of the assigned interventions, introducing a risk of performance bias.
D) As the study was not blinded, there is a risk of detection bias in outcome assessment, especially since visual acuity assessment may be subjective to some extent.
E) There was notable attrition: only patients with available one-year follow-up in phase 2 were analyzed; no imputation or sensitivity analysis for missing data was performed.
F) The study reports pre-specified outcomes (visual acuity at specified intervals), and results appear to be reported as planned.
G) There may be additional biases due to lack of blinding and attrition. Baseline characteristics were well-matched, but other unmeasured confounding cannot be excluded.

Pediatric Eye Disease Investigator Group (2003)
A) Both studies were described as randomized clinical trials. The randomization process was appropriately performed and described as central randomization, which minimizes selection bias.
B) Allocation concealment was maintained by central randomization and was unlikely to be predicted by the enrolling investigators, reducing the risk of selection bias.
C) Due to the nature of the interventions (atropine drops vs. patching), it was not possible to mask participants, parents, or treating personnel. This introduces a risk of performance bias, especially related to subjective outcomes such as adherence or parental reporting.
D) Visual acuity testing was standardized and performed by certified testers following the Amblyopia Treatment Study (ATS) protocol. Although masking of outcome assessors is not explicitly described in every section, prior PEDIG studies typically employed masked testers, and the outcome (visual acuity) is objective and less susceptible to bias.

E) Both articles clearly describe how patients who did not meet eligibility criteria or who withdrew before follow-up were excluded from the final analysis. Attrition rates were low and balanced across arms. Pre-specified criteria for including patients in the analysis were used, and intention-to-treat principles were followed as closely as possible.
F) All pre-specified primary and secondary outcomes were reported, including subgroup and compliance analyses. The protocol and outcomes are available, and reporting appears to be complete.
G) No other significant biases were identified. The studies were multicenter and supported by the National Eye Institute, with detailed methods, standardized protocols, and high external validity. Compliance was measured, but parent-reported compliance may introduce some bias, which is an unavoidable limitation of the design.

<u>Pediatric Eye Disease Investigator Group (2005)</u>
A) Randomization performed using a permuted-blocks design on a centralized web platform. Equal probability assignment to each group.
B) Allocation was implemented centrally via the study website and was concealed from investigators until assignment, minimizing bias.
C) Parents and children were aware of assigned activity, but group assignment was unlikely to bias the performance of prescribed activities.
D) Visual acuity testing was performed by study-certified testers using standardized protocols, but outcome assessors were not masked. The authors acknowledge that masking was not implemented, but argue that systematic bias is unlikely due to protocol standardization and multicenter design.
E) High completion rates for visual acuity outcome visit (91%). Missing data and withdrawals were balanced between groups.
F) All primary and secondary outcomes described in the methods are reported. No evidence of selective outcome reporting.
G) No other important sources of bias identified. Baseline characteristics were similar between groups.

<u>Pediatric Eye Disease Investigator Group (2008)</u>
A) Randomization was performed centrally using a permuted blocks design stratified by site and amblyopic eye visual acuity.
B) Central randomization through the PEDIG website ensured allocation concealment.
C) Due to the nature of the interventions (patching vs. atropine drops), participants and clinical personnel could not be blinded, introducing a risk of performance bias.
D) Primary outcome (visual acuity) was assessed by certified examiners who were masked to treatment group for 97% of the 17-week primary outcome assessments
E) The primary outcome was completed by 89% of participants (88/95 atropine, 84/98 patching), and missing data were similar between groups. Analyses followed the intention-to-treat principle.
F) The study was well powered, with balanced baseline characteristics and appropriate handling of protocol deviations.
G) The study was well powered, with balanced baseline characteristics and appropriate handling of protocol deviations.

<u>Pediatric Eye Disease Investigator Group (2009)</u>
A) Randomization was conducted using a computer-generated permuted block design, stratified by site, after eligibility was confirmed, reducing the risk of selection bias.

B) Allocation to treatment groups was performed centrally through the PEDIG website after eligibility confirmation, ensuring concealment from enrolling investigators.
C) Due to the nature of the interventions (plano lens vs. full hypermetropic correction), masking of participants and parents was not possible, as glasses differed visibly.
D) Visual acuity at the primary 18-week endpoint was assessed by masked (blinded) examiners for 99% of patients, minimizing detection bias.
E) Follow-up was high and well-balanced between groups: 93% (atropine-only) and 98% (atropine+plano) completed the 18-week outcome. Missing data unlikely to bias results; intent-to-treat analysis.
F) The study protocol was registered (ClinicalTrials.gov NCT00315302), primary and secondary outcomes were reported as planned, with full results provided.
G) Baseline characteristics were well balanced, and analysis adjusted for minor baseline imbalances. No significant protocol deviations. Treatment adherence was high in both groups.

Pediatric Eye Disease Investigator Group (2010)
A) Participants were randomly assigned using a permuted block design, stratified by age group and study site. Randomization procedures were adequately described and centrally administered.
B) Allocation was concealed through centralized randomization and stratification, preventing foreknowledge of group assignment by investigators and participants.
C) Blinding of participants and parents was not possible due to the nature of the interventions (patching vs. Bangerter filter). This could potentially introduce performance bias, particularly in parent-reported outcomes (e.g., compliance, Amblyopia Treatment Index). However, the main outcome (visual acuity) is objective and less likely to be influenced.
D) At the primary 24-week outcome visit, visual acuity was measured by a study-certified examiner masked to treatment allocation for 96% of examinations (95% in Bangerter group; 98% in patching group), minimizing detection bias for the main outcome.
E) Completion rates for the primary outcome were high and balanced (91% in both groups). Reasons for withdrawal or missing data were reported, and intention-to-treat analysis was performed. No evidence of differential attrition between groups.
F) The study protocol was registered (ClinicalTrials.gov NCT00525174) and available online. Primary and secondary outcomes were clearly specified and reported as planned. No evidence of selective reporting.
G) Compliance was assessed through parental diaries and investigator judgment, which may be subject to reporting bias (particularly as parents and children could not be masked). No objective compliance monitoring (e.g., occlusion dose monitors, filter sensors) was used. Some variability in effect may be attributable to differential compliance, especially in the Bangerter group.

Pediatric Eye Disease Investigator Group (2013)
A) Participants were randomized using a permuted block design stratified by site. The process was centrally administered, and procedures are well-described.
B) Allocation was performed centrally (via PEDIG website), preventing investigators from predicting assignment.
C) Due to the intervention (2 vs. 6 hours of patching), participants and parents could not be masked, which may introduce performance bias, particularly for subjective outcomes like compliance reporting. However, the primary outcome (visual acuity) is objective and less likely to be influenced.

D) The 10-week primary outcome visual acuity was assessed by a masked, study-certified tester for 97% of examinations (98% in the 6-hour group, 96% in the 2-hour group), minimizing risk of detection bias for the main outcome.
E) Completion rates for the primary outcome were very high (97–99% in both groups), and intention-to-treat analysis was performed. Reasons for missing data were minimal and balanced between groups.
F) The protocol was registered (ClinicalTrials.gov NCT00945100) and made publicly available. All pre-specified primary and secondary outcomes were reported. No evidence of selective reporting was found.
G) Compliance was assessed by investigator judgment and parent-reported diaries, which may be subject to reporting bias as parents and children were not masked. No objective occlusion dose monitoring was used. The possibility of continued improvement in some children labeled as "stable" at randomization (potential misclassification of stability) was discussed and appears evenly distributed.

Pediatric Eye Disease Investigator Group (2015)
A) Central randomization was performed via the PEDIG website using a permuted block design stratified by site and by atropine regimen. The process is clearly described, minimizing risk of selection bias.
B) Allocation was concealed by central randomization. Investigators could not foresee assignment.
C) Participants, parents, and clinicians could not be blinded due to the nature of the intervention (plano lens in spectacles vs. standard correction), introducing potential performance bias, especially for subjective outcomes (e.g., compliance). However, the primary outcome (visual acuity) is objective and less susceptible.
D) The primary outcome (10-week amblyopic-eye visual acuity) was assessed by a study-certified, masked examiner for 99% of participants, reducing risk of detection bias for the primary endpoint.
E) Nearly all participants completed the primary outcome (10-week) visit. Only one participant in the atropine-only group missed the analysis window; reasons were unrelated to study arm, and intention-to-treat was followed.
F) Study protocol was available publicly (PEDIG website). Primary and secondary outcomes were reported as pre-specified. No evidence of selective reporting.
G) The study was underpowered (recruitment was stopped early at less than half the planned sample size), which increases imprecision and the likelihood of a type II error. Compliance was investigator-judged and based on parent diaries, not objectively measured. The open-label nature could influence adherence or reporting.

Polat et al. (2004)
A) The study is described as "prospective, randomized, masked, controlled." Patients were allocated to intervention or control arms. Although the exact method (e.g., computer-generated sequence) is not specified, the use of randomization is clear and there is no evidence of bias in sequence generation.
B) No details are provided about how allocation to treatment or control was concealed. It is not clear if investigators enrolling patients could foresee upcoming assignments.
C) The text mentions the study was "masked," but does not clarify if participants and treating personnel were blinded to group allocation. The intervention (perceptual learning training) may make blinding difficult.

D) Visual acuity measurements were performed by "clinicians who were blinded to the subgroup (treatment or control) to which the examinee had been allocated." Thus, outcome assessment was likely blinded.
E) All randomized participants were included in the analysis. Reasons for drop-out are not detailed, but there are no significant losses to follow-up reported that would introduce bias.
F) Outcomes were pre-specified (visual acuity, contrast sensitivity, spatial interactions) and all main outcomes are reported in the results. There is no evidence of selective reporting.
G) Two authors had a financial interest in the company related to the perceptual learning method (NeuroVision, Inc.), representing a potential conflict of interest. Also, while the placebo group had a similar structure, the precise equivalence of treatment duration and attention is not fully detailed. Sample sizes in control groups are small.

<u>Poltavski et al. (2025)</u>
A) Random assignment to eye patching or video game groups was performed; randomization process is described in the methods.
B) Allocation was performed by unmasked staff, but the sequence itself was not predictable to clinicians, and randomization was done after eligibility confirmation, reducing selection bias.
C) Participants and therapists could not be blinded due to the nature of the interventions (patching vs. video game). This is a common limitation in behavioral/device interventions
D) Visual acuity assessments were performed by optometrists (“blinded examiners”) masked to treatment allocation at all follow-ups, reducing risk of detection bias.
E) 22.5% attrition (49 enrolled, 40 completed). Reasons for dropout were largely related to the COVID-19 pandemic, but differential attrition between groups is not specified in detail.
F) Outcomes were reported as specified (non-inferiority in visual acuity improvement at 12 weeks), and results were fully reported for completers.
G) The study had a relatively small sample size and some selection bias is possible due to volunteer participants. Compliance was monitored via home logs, but no objective compliance tracking.

<u>Proudlock et al. (2024)</u>
A) Participants were randomized centrally using a computer-generated sequence with block randomization and stratification by amblyopia type and severity.
B) Allocation was performed using a secure online randomization service (Sealed Envelope), inaccessible to recruiting clinicians until group assignment.
C) Participants, clinicians, outcome assessors, and statisticians were not masked to treatment allocation due to the nature of the interventions (extended glasses use vs. patching).
D) Outcome assessors were not masked, and visual acuity testing could be influenced by lack of masking, though objective visual acuity protocols (logMAR) were used.
E) Attrition was somewhat higher in the EOT group (13%) than early patching (5%), mainly due to loss to follow-up and withdrawal. The primary analysis followed a modified ITT approach; sensitivity analyses with imputation yielded consistent results.
F) The protocol was registered (ISRCTN51712593) and the primary/secondary outcomes were reported as planned.
G) Some missing adherence data due to technical issues with electronic monitors. The multicenter design and stratified randomization help minimize center-related biases.

Repka et al. (2007)
A) Participants were randomized to treatment groups in a controlled trial, with randomization procedures overseen by the Pediatric Eye Disease Investigator Group (PEDIG).
B) Allocation concealment is not explicitly described, but as a PEDIG multicenter RCT, standard protocols likely applied; nonetheless, not detailed in the report.
C) Due to the obvious nature of interventions (patching vs. atropine drops), blinding of participants and clinicians was not possible, resulting in high risk for performance bias
D) Refractions were performed using cycloplegic retinoscopy by masked examiners in PEDIG protocols, but the masking for this secondary outcome (refractive error) is not fully detailed.
E) Only 282/419 (67%) completed the 2-year refraction, raising potential for attrition bias, though the baseline characteristics were reported as similar between completers and non-completers.
F) The primary and secondary outcomes were reported as planned.
G) Some children changed or discontinued assigned treatments after 6 months, and data on actual duration of treatment beyond 6 months were not available, introducing possible bias.

Roy et al. (2023)
A) The study was described as "prospective, randomized" with allocation into two groups; randomisation process is explicitly mentioned.
B) The method for concealing allocation (e.g., sealed envelopes, centralized assignment) is not described in the abstract.
C) Due to the nature of interventions (video game vs. patching), masking of participants, parents, and therapists is not feasible.
D) The article does not mention whether outcome assessors (measuring VA, contrast, stereoacuity) were masked to treatment group.
E) The article does not report loss to follow-up or exclusions; results are given for all randomized participants (n=55).
F) All main planned outcomes (distance/near vision, contrast sensitivity, stereoacuity) are reported in the abstract.
G) No other apparent sources of bias; baseline characteristics similar; interventions and outcomes appropriate for the research question.

Sharif et al. (2019)
A) Randomization and assignment performed using research randomizer software; method clearly described.
B) Placebo capsules identical in appearance; assignment by third party (pharmacy). Double-blind.
C) Double-blind: both participants and clinical staff were masked to allocation.
D) Outcomes (visual acuity, etc.) were assessed by an examiner blinded to treatment allocation.
E) Flow diagram provided; dropouts reported (1/21 fluoxetine, 4/19 placebo), reasons described; analysis on all completers (20/15)
F) Groups well balanced at baseline; adherence assessed; funding source declared, no conflicts of interest.
G) Groups well balanced at baseline; adherence assessed; funding source declared, no conflicts of interest.

Singh et al. (2017)
A) The study states that participants were “randomly allocated into two groups,” but no details are provided regarding the method of randomization (e.g., computer-generated, blocks).

B) There is no description of allocation concealment or whether group assignment was concealed from enrolling investigators or participants.
C) The intervention (video game play vs. no video game) likely could not be blinded for participants, and there is no mention of blinding of staff delivering the intervention.
D) There is no mention of whether the examiners assessing visual acuity, stereoacuity, or contrast sensitivity were masked to group allocation.
E) The study appears to report outcomes for all randomized participants; no information suggests differential loss to follow-up or attrition.
F) All specified outcomes (BCVA, stereoacuity, contrast sensitivity at specified time points) are reported as planned.
G) Baseline characteristics (age, baseline VA) appear balanced between groups; no other sources of bias are apparent from the description.

Stanković & Milenković (2007)
A) Patients were "randomly assigned" to treatment groups; randomization is described, but the method for sequence generation is not specified in detail.
B) The process for allocation concealment is not described (e.g., no mention of sealed envelopes or centralized randomization), so risk here is unclear.
C) Due to the nature of the interventions (continuous vs alternating full-time occlusion), blinding participants and treating staff was not feasible.
D) It is not explicitly stated whether outcome assessors (those measuring visual acuity, PVEP, etc.) were blinded to group assignment, so detection bias cannot be excluded
E) Attrition is reported (2/53 patients excluded after developing occlusion amblyopia and crossing over), and the reasons for exclusion are provided. Overall attrition is low.
F) Primary and secondary outcomes (visual acuity, crowding difficulties, PVEP, compliance) are all reported in detail, with statistical results and tables included.
G) Compliance was assessed by parental/examiner scoring, which is subjective and prone to bias. There is potential performance bias and reporting bias regarding compliance.

Tejedor & Ogallar (2008)
A) Randomization was done using a computer-generated sequence of random numbers, after stratification by cause of amblyopia.
B) The method of allocation concealment is not described; potential risk that group assignment could be anticipated.
C) Blinding of participants and personnel is not reported; both treatments are distinct and likely recognizable by participants, but effect on outcomes (VA) is probably minimal.
D) Visual acuity examiners were masked to treatment group. Blinding was confirmed in 87% (atropine) and 90.6% (optical penalization) of cases.
E) Low and similar loss to follow-up in both groups (about 9-11%). The study used intention-to-treat analysis and reported reasons for dropout.
F) All pre-specified primary (visual acuity, interocular difference) and secondary (stereoacuity) outcomes are reported; results match the protocol and stated aims.
G) Compliance was difficult to monitor, especially in the optical penalization group (peeking over glasses); this could introduce performance bias, but its impact seems limited.

Tejedor & Gutiérrez-Carmona (2023)

A) Random allocation via computer-generated random number table, performed by a study committee.

B) Randomization and assignment by committee; not explicitly detailed if allocation sequence was concealed, but process implies concealment.

C) Investigators masked to treatment assignment; families and children likely aware of bifocal use, but primary outcome is objective (VA).

D) Outcomes (visual acuity, etc.) measured by masked investigators.

E) 1 lost to follow-up per group (19/20 and 16/17 analyzed); flow detailed, no evidence of differential dropout or bias.

F) All prespecified primary and secondary outcomes reported; methodology and results are consistent.

G) Baseline characteristics balanced, compliance monitored, adverse events reported, no significant COI or funding bias identified.

Uttamapinan et al. (2024)

A) Used computer-generated stratified permuted block randomization with varying block size and sealed envelopes.

B) Allocation sequence was concealed using sealed envelopes.

C) Participants, caregivers, and investigators were not masked due to the nature of the intervention (app use vs logbook).

D) Compliance was self-reported by caregivers in both groups; BCVA measured by ophthalmic nurse, but masking to group assignment not specified (likely unblinded).

E) Only one participant lost to follow-up at 3 months (very low attrition, and reported).

F) Outcomes were pre-specified (compliance and BCVA) and all reported as planned.

G) Compliance measured by subjective caregiver report (not by objective monitor), possible Hawthorne effect, potential for expectation bias in the intervention group.

Wang et al. (2016)

A) Randomization was performed using sealed envelopes opened by the child or their parents. The envelopes were pre-prepared and allocation was by chance.

B) Assignment was concealed in sealed, opaque envelopes until the moment of allocation, minimizing selection bias.

C) Blinding was not possible: participants and families were aware of the treatment (IO glasses vs. patching), and investigators were not masked to group assignment.

D) All randomized participants were included in the primary outcome analysis (intent-to-treat). Dropout and missing data were balanced and reported.

E) All randomized participants were included in the primary outcome analysis (intent-to-treat). Dropout and missing data were balanced and reported.

F) All outcomes described in the methods (visual acuity, compliance, adverse events) were reported as planned in the results.

G) Baseline characteristics were similar; study followed a pre-registered protocol (clinicaltrials.gov NCT01973348). No other sources of bias identified.

Wang et al. (2021)

A) Randomization sequence was generated using statistical software by an independent statistician; stratified by age.

B) Allocation details were placed in sequentially numbered, opaque, sealed envelopes, which were opened only after baseline assessments—ensuring concealment.
C) Due to the nature of the intervention (atropine + patching vs patching), participants and parents were not blinded, which may introduce performance bias.
D) The visual acuity examiner was masked to treatment allocation at all visits, minimizing detection bias for the primary outcome.
E) Attrition was low and balanced between groups (91% and 87% completed 6-month follow-up), and intention-to-treat analyses with multiple imputation for missing data were conducted.
F) All pre-specified outcomes (primary and secondary) were reported; protocol and statistical analysis plan were available.
G) Baseline characteristics were well balanced, adherence was monitored, and the trial was registered (Chinese Clinical Trial Registry: ChiCTR1800018663). No other major sources of bias were identified.

Wu et al. (2010)
A) The study used a computer-generated randomization sequence.
B) Allocation was concealed; randomization sequence was kept by an investigator not involved in assignments, ensuring concealment.
C) Due to the nature of the interventions (patching vs. patching + telescope), participants, caregivers, and investigators could not be masked.
D) Outcome assessment was not masked; visual acuity measurement and data analysis were performed with knowledge of group assignment.
E) All randomized patients completed the study protocol and outcome measurements (no attrition).
F) All pre-specified outcomes, including secondary endpoints, were reported in the results section.
G) Very small sample size (n=15, pilot study), and baseline differences in amblyopia type between groups (e.g., more mixed and strabismic cases in telescope group), which may introduce bias and limit generalizability.

Wygnanski-Jaffe et al. (2023)
A) Randomization was performed centrally using permuted blocks, stratified by site, and described in detail.
B) Allocation concealment was achieved using secure electronic web-based software; allocation sequence was not accessible to investigators before assignment.
C) Participants and families could not be fully blinded due to the nature of the interventions (device vs. patching), but evaluators and outcome assessors were masked to treatment group at all follow-ups.
D) Primary outcome assessments (visual acuity and stereoacuity) were performed by masked evaluators, reducing detection bias.
E) Loss to follow-up was minimal and balanced between groups; primary analysis was performed on a modified intention-to-treat (mITT) population, with sensitivity analyses on ITT and per-protocol sets.
F) The trial was prospectively registered (ClinicalTrials.gov NCT05185076; MOH_2020-08-10_009227) and all prespecified primary and secondary outcomes were reported.
G) Adherence was objectively measured in the binocular group (device logs) but relied on subjective guardian diaries in the patching group, which may slightly overestimate patching compliance.

Wygnanski-Jaffe et al. (2025)

A) The study used a permuted block randomization design stratified by site with a 1:1 allocation ratio, which is an appropriate method to ensure adequate sequence generation.

B) Allocation was managed centrally, and there is no indication that investigators could predict group assignments, minimizing the risk of allocation bias.

C) Due to the nature of the interventions (eye patching vs. digital binocular treatment at home), it was not possible to blind participants or personnel, which may have affected adherence and subjective outcomes.

D) The study reports that outcome assessors were masked to group assignments, reducing the risk of detection bias in outcome measurements.

E) Attrition was low and similar between groups. Both modified intention-to-treat (mITT) and per-protocol (PP) analyses were performed, reducing the risk of attrition bias.

F) All prespecified primary and secondary outcomes were reported, including subgroup analyses and adherence data.

G) No other significant sources of bias were identified. The study was registered, and methods were clearly described

Xiao et al. (2022)

A) Randomization was conducted centrally using a secure, web-based computer program with permuted block design, stratified by site, and varying block sizes (2 or 4). This approach ensures an adequately random sequence.

B) Allocation was concealed through the use of web-based software, keeping the randomization sequences hidden from site investigators and participants until assignment. This prevents foreknowledge of allocation.

C) Blinding of participants and personnel administering the intervention was not possible, as the nature of the intervention (dichoptic therapy device versus glasses alone) is readily apparent. Placebo packaging was used for the control group, but this would not fully prevent participants from knowing their group.

D) Examiners who measured primary outcomes (visual acuity) were masked to treatment assignment at all follow-up visits. Instructions were given to participants to avoid discussing their treatment with examiners. No unmasking events occurred. Visual acuity measurement is objective, minimizing detection bias.

E) 90 out of 105 participants (86%) completed the 12-week primary outcome visit. Reasons for missing data were described and appeared balanced between groups. Sensitivity analyses, including multiple imputation, did not alter the main results.

F) The trial was prospectively registered (ClinicalTrials.gov NCT03608150), and all prespecified primary and secondary outcomes were reported. The statistical analysis plan was followed, and protocol deviations were minimal.

G) Several authors were employees, officers, or equity holders in the sponsor company (Luminopia, Inc.), which also funded the trial and was involved in the study design, conduct, analysis, interpretation, and manuscript preparation. Although this is fully disclosed and does not affect the internal validity, it introduces potential conflict of interest that should be considered when interpreting results.

Yuan et al. (2021)

A) Participants were randomized into two groups (AFG or patching); the article clearly states the use of random assignment.

B) The method of allocation concealment is not described in detail. It is not clear whether group allocation could have been foreseen by researchers or participants.
C) Blinding was not possible due to the obvious nature of the interventions (AFG glasses vs. patching), so neither participants nor treating clinicians were blinded.
D) No information provided about whether outcome assessors were blinded; visual acuity and stereoacuity are objective but still susceptible to bias if not masked.
E) All randomized participants completed follow-up and were included in the analysis; there is no mention of dropouts or missing data
F) All stated outcomes (BCVA, CSF, stereoacuity) were reported as planned. No evidence of selective outcome reporting.
G) Small sample size (pilot study), only children with anisometropic amblyopia included, short follow-up (12 weeks), and potential performance bias due to unblinded design.

Zhao et al. (2010)
A) The study used a computer-generated randomization list.
B) The method for allocation concealment was not detailed (no mention of sealed envelopes or electronic system).
C) Participants and parents could not be masked due to the nature of interventions (acupuncture vs. patching).
D) Visual acuity examiners were masked to group allocation; testing and acupuncture were performed on different floors, and families were instructed not to disclose the assigned group.
E) Low and balanced attrition between groups (acupuncture 2/43; patching 3/45 at 25 weeks), with reasons for dropout reported and analyzed.
F) All pre-specified primary and secondary outcomes are reported in the article.
G) No evidence of other major sources of bias. Funding and potential conflicts of interest (patent application) were disclosed.

Zhu et al. (2023)
A) The study describes randomization using a random number generator and sealed, sequentially numbered envelopes, supporting adequate sequence generation.
B) Allocation was concealed using sealed, sequentially numbered envelopes, opened only after eligibility confirmation, minimizing allocation bias.
C) The intervention (binocular therapy with 3D movies vs. patching) makes blinding of participants and personnel impractical; thus, there is a high risk of performance bias.
D) Visual function measurements were conducted by study-certified examiners who were masked to treatment allocation, reducing detection bias.
E) Attrition was low and balanced (3 withdrawals out of 35 enrolled; 86% and 100% follow-up in intervention and control groups, respectively), with clear reasons for dropouts.
F) All pre-specified primary and secondary outcomes appear to be reported fully, with sufficient detail in both text and supplementary materials.
G) The small sample size, short duration of follow-up, and reliance on parental report for patching compliance could introduce additional bias; potential for compliance bias.
