# Supplementary material for: Systematic Review and Meta-Analysis of RCTs on Efficacy of Conventional vs. Emerging Treatments for Amblyopia
Source: Life (Basel). 2026 Jan 28;16(2):222. doi: 10.3390/life16020222 (PMC12942344; doi:10.3390/life16020222)
Supplement: Supplementary file 1 [file life-16-00222-s001.zip › Additional file S5.pdf]

Additional file S5. Reasons for full-text exclusion

Reason for exclusion	Description
Non-comparative design	Studies without a randomized comparative design or appropriate control group
Inadequate or incomplete data	Missing or insufficient outcome data for quantitative synthesis
Lack of extractable outcomes	Outcomes not reported in an extractable format
Overall high risk of bias	Overall high risk of bias according to predefined criteria (Section 2.6)
