# Supplementary material for: Systematic Review and Meta-Analysis of RCTs on Efficacy of Conventional vs. Emerging Treatments for Amblyopia
Source: Life (Basel). 2026 Jan 28;16(2):222. doi: 10.3390/life16020222 (PMC12942344; doi:10.3390/life16020222)
Supplement: Supplementary file 1 [file life-16-00222-s001.zip › Additional file S6.pdf]

Additional File S6. Baseline characteristics of the 65 included studies.

Author (Year)	Country/ Region	Study Design	Sample Size	Mean Age	Amblyopia Type(s) Included	Treatment(s) Compared	Treatment Duration (weeks)	Amblyopia Diagnostic Criteria	COI
Agervi et al. (2013)_Bangerter	Sweden, Norway	RCT	33 (Spectacles) / 32 (Spectacles +Bangerter)	4.5 (Spectacles)/ 4.5 (Spectacles+Bangerter)	Anisometropic amblyopia only	Spectacle correction alone vs. Spectacle correction + Bangerter filter (fellow eye)	104	Unilateral amblyopia: ≥2-line difference on Lea symbols chart; VA amblyopic eye ≥0.3 logMAR, fellow eye ≤0.1 logMAR; anisometropia = ≥1.0 D spherical or ≥1.5 D cylindrical difference	No
Agervi et al. (2013)_S + ADP	Sweden	RCT	20 (alternate-day) / 20 (daily patching)	4.3 (Alt) / 4.4 (Daily)	Strabismic and mixed (Strabismic + Anisometropic)	Spectacles + alternate-day patching vs. spectacles + daily patching (6 days/week)	104	≥2-line interocular difference on Lea symbols; VA ≥0.3 logMAR in amblyopic eye and ≤0.1 logMAR in fellow eye	No
Bhartiya et al. (2002)	India (New Delhi)	RCT	19 (Levodopa) /21 (Placebo)	10.6 (Levodopa) / 11.3 (Placebo)	Strabismic and anisometropic amblyopia	Levodopa-Carbidopa + occlusion vs. Placebo + occlusion	12	LogMAR visual acuity (ETDRS chart), clinical criteria	No
Chen et al. (2008)	Taiwan	RCT	26 (perceptual learning)/ 27 (patching)	17.3(perceptual learning)/ 13 (patching)	Anisometropic (children + adults; no strabismus**)**	Perceptual learning (Gabor patch) vs. Patching	PL: avg. 29.5 h (48 sessions); Patch: avg. 522 h (37.3 wks)	Anisometropia (≥1.0 D difference), IOD ≥0.2 logMAR; no strabismus; baseline VA 0.2–0.8 logMAR; no prior patch/atropine	No
Chen et al. (2025)	China (Shanghai)	RCT	20 (gamified binocular) / 22 (patching)	5.60 (binocular)/ 6.09 (patching)	Refractive (93%), strabismic (4.7%), combined (2.3%)	Gamified binocular treatment (Vision Planet System, 2 × 30 min/day) vs. patching (90 min/day)	24	Age 4–8 yrs; VA difference ≥2 lines; amblyopic eye VA 20/32–20/200; refractive, strabismic, or combined amblyopia	No
Dadeya & Dangda (2016)	India	RCT	20 (patching + video games)/ 20(patching)	6.03 ± 1.14 years (overall)	Unilateral amblyopia	(A) Full-time patching alone (B) Full-time patching + TV video games (12 × 0.5h weekly sessions)	12	Age 4–7 years; Unilateral amblyopia; BCVA in AE: 1.0–0.6 logMAR after 6 wks optimal correction	No
Dahlmann-Noor et al. (2024)	UK	RCT	11(BBV)/ 9 (Control)	4.8(BBV)/ 5.1(Control)	Anisometropic, strabismic, mixed (combined)	Balanced binocular viewing (BBV, 1h/d) vs. Standard care (patching 2–6h/d or atropine)	16	Unilateral amblyopia (3–8 años), diferencia interocular de BCVA persistente ≥2 visitas separadas por ≥8 semanas, tras adaptación óptica si necesario	No
Elhusseiny et al. (2021)	USA (Boston)	RCT	11 (Full treatment)/	10 (Full treatment)/ 9	Unilateral anisometropic	(1) Virtual reality-based binocular therapy	8	Age 7–38 yrs; unilateral amblyopia (anisometropic, strabismic, or combined);	No

			9 (Sham-crossover)	(Sham-crossover)	and/or strabismic amblyopia; history of prior treatment failure	(2) Sham (placebo) then cross-over to active treatment (4 weeks sham + 4 weeks therapy)		BCVA amblyopic eye 20/40–20/200 despite previous treatment; stable VA ≥16 weeks prior to enrollment	
Evans et al. (2011)	UK	RCT	15 (IPS), 15 (Control)	32 (IPS), 30 (Control)	Strabismic (75%), anisometropic (25%), mixed	Intermittent Photic Stimulation (IPS) vs. placebo (CAM)	6	Unilateral amblyopia; VA better than 6/36 and worse than 6/9 in amblyopic eye; ≥1D hypermetropia or ≥2D astigmatism difference; no pathology; ≥10 yrs; no strabismus in first 2 years; up-to-date refractive correction	No
Foley-Nolan et al. (1997)	Ireland	RCT	18 (Atropine)/ 18 (Patching)	5.5 years (both groups)	Strabismic and anisometropic	(A) Atropine penalisation (1% daily to sound eye)<br>(P) Occlusion of sound eye (full-time/age-adapted)	Atropine: mean 7.2 mo (1–12)<br>Patching: mean 4.3 mo (2–9)	Initial VA ≤6/18; Strabismic/anisometropic amblyopia	No
Fresina et al. (2008)	Italy	RCT	32 (CDP-choline+patching)/ 29 (Patching alone)	5.9 (CDP-choline+patching)/ 6.2 (Patching alone)	Anisometropic, strabismic	(1) Oral CDP-choline (800–1200 mg/day) + 2h patching/day<br>(2) 2h patching/day only	30 days treatment, 90-day follow-up (i.e. 60 days after cessation)	Age 5–10 yrs; mono-ocular; ≥2 lines interocular difference or ≤20/30 (Snellen) in amblyopic eye	No
Gao et al. (2018)	Multicenter (New Zealand, Australia, Canada, Hong Kong)	RCT	56 (active binocular game) / 59 (placebo game)	22.1 (active), 21.0/ (placebo)	Anisometropic, strabismic, mixed	Binocular dichoptic video game vs. placebo (non-dichoptic) game (both 1 h/day at home)	6	Age ≥7 y; unilateral amblyopia (VA 0.30–1.00 logMAR in amblyopic eye, fellow eye ≤0.10 logMAR); stable VA after optical correction; anisometropia/strabismus/mixed	No
Garcia-Romo et al. (2018)	Spain	RCT	5 (OCL) / 5 (Patching)	40 (range 20–50)	Anisometropic, strabismic, and mixed	Prosthetic occluding contact lenses vs. eye patching	52	≥0.1 logMAR in amblyopic eye; ≥0.2 logMAR interocular difference; presence of anisometropia or strabismus	No
Herbison et al. (2016)	UK	RCT	24 (I-BiT DVD) / 26 (I-BiT game) / 25 (non-I-BiT game)	5.9 (I-BiT DVD)/ 6.0(I-BiT game) / 5.6(non-I-BiT game)	Strabismic, Mixed, Anisometropic	I-BiT DVD vs I-BiT game vs Non-I-BiT game (placebo dichoptic with same stimuli to both eyes)	6 (1 session/week × 30 min)	Age 4–8 years; ≥0.20 logMAR interocular VA difference; strabismic, anisometropic or mixed; no current improvement with patching	No
Hernández-Andrés et al. (2025)	Spain	RCT	17 (perceptual learning)/ 17 (patching+vision	perceptual learning (7.4)/ patching+vision therapy (7.3)/patching (5.8)	Anisometropic, Strabismic, Mixed	(1) Patching 2h/día; (2) Monocular perceptual learning (Smiling Face game); (3) Patching 2h + Vision Therapy; Control	12	Monocular amblyopia; refraction ≤±9D; IOD-VA ≥2 lines; strabismus ≤35Δ; no ocular cx (except strabismus); complete refractive adaptation	No

			therapy)/ 18 (patching)						
Holmes et al. (2016)	USA (PEDIG multicenter)	RCT	190 (Binocular game) / 195(Patching)	8.5 (both groups)	Strabismic, anisometropic, or combined amblyopia	Binocular iPad game (1 hr/day) vs. patching (2 hr/day)	16	Age 5–<13 yrs; amblyopic-eye VA 20/40–20/200 (mean 20/63); due to strabismus, anisometropia, or both; prior treatment allowed (stratified randomization)	No
Holmes et al. (2019)	USA, Canada (multicenter)	RCT	69 (Binocular) / 69 (Patching)	9.6 (Binocular) / 9.6 (Patching)	Strabismic, anisometropic, or combined	Binocular Dig Rush iPad game + spectacles vs. spectacles alone	8	Age 7–12 yrs; amblyopic eye VA 33–72 letters (approx. 20/200–20/40); ≥16 weeks prior optical treatment or VA stability ≥8w; interocular difference ≥3 logMAR lines	NIH NEI (EY011751, EY018810); no conflicts reported
Huang et al. (2022)	China	RCT	18 (AMTP + patching)/20 (Patching)	5.44 (AMTP + patching)/5.48 (Patching)	Anisometropic amblyopia only	(1) Asynchronous 3D movie training + patching (2) Patching alone	12	Age 4–8; anisometropia ≥1.5D SE or ≥1.0D cylinder; BCVA 0.2–1.0 logMAR in AE; ≥3 months stable correction; <4 weeks prior patching	No
Huttunen et al. (2018)	Finland, Estonia	RCT	22 (Fluoxetine) / 20 (Placebo)	38.5 (fluox)/ 36.4 (placebo)	Moderate/severe amblyopia due to anisometropia (myopic or hyperopic) or congenital esotropia	Fluoxetine 20 mg/day + patching + perceptual training vs. placebo + patching + perceptual training	22	Age 18–60; moderate (0.3–0.6 logMAR diff.) or severe (>0.6 logMAR diff.); amblyopic eye VA 0.30–1.10 logMAR; dominant eye VA ≤0.10 logMAR; untreated last 2 years	No
Jost et al. (2022)	USA (Texas)	RCT	32 (Dichoptic movies), 33 (Patching)	6.0 (movies)/ 6.1(patching)	Strabismic, anisometropic, or combined mechanism	Streaming at-home contrast-rebalanced dichoptic movies vs. patching (2h/day)	6	Age 3–7 years; amblyopic eye BCVA 0.2–0.8 logMAR; fellow eye −0.1 to 0.2 logMAR; interocular VA diff. ≥0.2 logMAR; prior glasses ≥8 wks; stable VA	No
Jost et al. (2024)	USA (Dallas, TX)	RCT	17 (Dichoptic cartoon) / 17 (Patching)	5.3 (Dichoptic) / 5.1(Patching)	Strabismus, anisometropia, combined	Contrast-rebalanced dichoptic cartoons (Q Pootle 5) on Nintendo 3DS XL, 4h/week (home-based, 2 weeks) + 2 extra weeks for cartoon group	2	(1) Amblyopic eye BCVA 0.20–0.70 logMAR; (2) Fellow eye BCVA 0.1–0.3 logMAR; (3) Interocular diff ≥0.3 logMAR; (4) Strabismus <5Δ after correction; (5) ≥8 weeks glasses wear with stable BCVA	No
Kadhum et al. (2024)	Netherlands	RCT	16 (Gaming / 17 (Occlusion)	5.8 (gaming) / 4.9 (occlusion)	Anisometropic (28), Strabismic (3), Combined (2)	Dichoptic VR gaming (1h/week supervised) vs. Monitored occlusion (2h/day)	24	Interocular VA difference ≥ 0.2 logMAR after 16-week refractive adaptation; age 4–12 years; excluded >30PD strabismus	No
Kelly et al. (2016)	USA (Dallas, TX)	RCT	14 (Binocular)/ 14 (Patching)	6.7 years overall (range 4.6–9.5)	Strabismic (32%), anisometropic (50%), combined-mechanism (18%)	Binocular iPad game (Dig Rush) vs. Patching (2h/day)	2 weeks each arm (4 weeks total, crossover)	Age 4–10 yrs; amblyopic eye BCVA 0.3–0.8 logMAR (20/40–20/125); fellow eye 0.1 logMAR (20/25) or better (0.2 logMAR for 4-year-olds); ≥0.3 logMAR (≥3 lines) interocular	Thrasher Research Fund, NIH NEI (EY02313); no

								difference; strabismus aligned to ≤4 prism diopters	conflicts reported
Khorrami-Nejad et al. (2024)	Iran	RCT	55 (CAM)/ 55 (Occlusion)	7 (CAM)/ 6.9 (Occlusion)	Unilateral amblyopia (anisometropic, others)	CAM therapy (30 min, 2×/sem) + occlusion vs. Passive occlusion therapy	12	Amblyopic children, no prior treatment, diagnosis based on interocular difference in CDVA (exact criterion not detailed in abstract)	No
Lagas et al. (2019)	New Zealand (Auckland)	RCT	7 (crossover, all participants received both arms)	33.4 (range: 19–47)	Strabismic, anisometropic, or mixed	Citalopram 20 mg/d + patching vs. Placebo + patching	2 + 2 (washout 2 wks)	Age ≥18 yrs; amblyopic eye VA ≥0.2 logMAR; fellow eye VA ≤0.0 logMAR; IOD ≥0.2 logMAR; strabismus/anisometropia ≥1.5 D	No
Leguire et al. (1993)	USA	RCT	14 (Levodopa/ Carbidopa)/ 6 (Placebo)	Range: 4–14	Anisometropic, strabismic, mixed, deprivational	Levodopa/Carbidopa (25/6.25 mg or 50/12.5 mg) vs. placebo + occlusion	0.03 (1 day)	Stable or difficult-to-treat amblyopia; VA in amblyopic eye ~20/121; occlusion applied during testing period	No
Leguire et al. (1998)	USA	RCT	7 (Levodopa+ Occlusion)/ 6 (Levodopa only)	9.1 (Levodopa+Occlusion)/8.7 (Levodopa only)	Anisometropic, strabismic, combined (inc. deprivation)	(1) Levodopa-carbidopa + part-time occlusion (3h/day) (2) Levodopa-carbidopa alone (no occlusion)	7 weeks + 4-week follow-up	Older children (7–12 yrs) with stable amblyopia, previously unresponsive to occlusion/penalization; refractive/cycloplegic confirmation; mean baseline VA ~20/116 (occlusion), ~20/90 (no occlusion)	No
Lin & Cai (2025)	China	RCT	74 ICL / 74 ICL + rTMS	28.6 (ICL) / 29.1 (ICL + rTMS)	Adult anisometropic amblyopia	ICL (posterior chamber lens) vs. ICL + rTMS	12	(1) Unilateral anisometropic amblyopia; (2) Age 18–50 years; (3) BCVA < 0.8 in one eye; (4) Interocular diff ≥1.50D; (5) No other ocular/systemic diseases; (6) Wore best correction ≥3 months with subnormal vision	No
Ma et al. (2024)	China	RCT	36 (Acu+Conv) / 38 (Conv)	36(Acu+Conv)/ 8.63(Conv)	Anisometropic (monocular)	1. Conventional (patching + visual stimulation)<br>2. Conventional + Acupuncture (BL1, BL2, GB20, GB37)	4	1) Monocular AA, 6–12 years<br>2) BCVA below normal for age or interocular difference ≥2 lines; SE ≥1.5 D, cyl ≥1.0 D<br>3) No ocular/neurological diseases<br>4) No treatment within last 3 months	No
Manh et al. (2018)	USA (multicente)	RCT	40 (Binocular) / 60(Patching)	14.3 years overall (13–<17 yrs)	Strabismic, anisometropic, or mixed mechanism	Binocular iPad game (1h/day) vs. Patching (2h/day)	16	Age 13–<17 yrs; amblyopic eye VA 20/40–20/200 (~20/63 mean); prior optical treatment/refractive adaptation required	No
Manny et al. (2022)	USA (multicente)	RCT	92 (Binocular)	5.7 (range 4–6) (both groups)	Anisometropic (63%), strabismic	Binocular Dig Rush iPad game + spectacles vs. spectacles alone	8	Age 4–<7 years; amblyopic eye VA 20/40–20/200; interocular VA difference ≥3 logMAR	No

			/ 90 (Patching)		(16%), combined (20%)			lines; ≥16 weeks prior optical treatment or VA stability ≥8w	
Meqdad et al. (2024)	Egypt	RCT	44 (VR) / 42 (Patching)	14.5 (VR) / 12 (Patching)	Anisometropic and mixed (moderate & severe)	VR Dichoptic Training (Vivid Vision) vs. Patching	10	VA ≤ 6/12 and interocular difference ≥ 0.2 logMAR; prior optical correction ≥12 weeks	No
Menon et al. (2008)	India (single center)	RCT	28 (Atropine) 29 (Patching)	13.75 (Atropine) 13.53 (Patching)	Anisometropic amblyopia (hypermetropic >1D)	(1) Full-time patching (2) Atropine penalization	24	Age 8–20 yrs; anisometropic hypermetropia >1D; interocular VA difference ≥3 logMAR lines; VA in sound eye 6/9; VA in amblyopic eye 6/12 to 6/60	No
Min et al. (2021)	China	RCT	15 (EFG) / 16 (Patching)	5.3 (EFG) / 6.4 (Patching)	Unilateral, mild–severe	Eyetronix Flicker Glass vs. Patching (2h/día)	12	Age 4–13, VA ≥0.3 (logMAR), diff ≥2 lines between eyes, CMAC 2011 criteria	No
Mirmohammadsadeghi et al. (2024)	Iran	RCT	29 (Fluoxetine) 26 (Placebo)	25.9 (Fluoxetine) 28.8 (Placebo)	Anisometropic, strabismic, combined	(1) Fluoxetine 20 mg/day + patching (2) Placebo + patching	12	Age >18 yrs; interocular VA difference ≥1 line; better eye VA >20/25; anisometropic: >1.5 D diff. (sphere/cylinder); strabismic: horizontal deviation <8D; no ocular/systemic disease	No
Mohamed et al. (2025)	Egypt	RCT	20 (part-time occlusion) / 20 (syntonic phototherapy)	10.7(occlusion); 18.2 (syntonic);	Refractive amblyopia (unilateral/bilateral; anisometropic, isoametropic)	Part-time occlusion vs. syntonic phototherapy	12	Refractive amblyopia; interocular BCVA difference ≥2 lines; no prior patching/atropine; ≥3 months in correction; exclusion of strabismus, ocular disease, pregnancy/lactation, history of seizures	No
Pang et al. (2021)	Hong Kong (single-center study)	RCT	12 (Active) / 11 (Placebo)	26 (Active) / 26 (Placebo)	Anisometropic, Strabismic, Mixed	Dichoptic video game vs. placebo video game	24	Aged ≥7 years; DVA in amblyopic eye ≤0.28 logMAR; interocular DVA difference ≥0.20 logMAR; stable VA with full correction; able to align nonius cross and complete fixation tasks	No
Pawar et al. (2014)	India	RCT	40 (Citicoline+ Patching) / 44 (Patching Only)	6.78 (Citicoline+Patching) / 6.68 (Patching Only)	Strabismic, anisometropic, combined	Citicoline + patching vs. patching alone	52	4–13 years old, clinical diagnosis, baseline workup incl. VA, refraction, exclusion of other pathology	No
Pediatric Eye Disease Investigator Group (2003)	USA, Canada	RCT	195 (Atropine) / 209 (Patching)	5.2 (Atropine) / 5.3 (Patching)	Strabismic, anisometropic, mixed	Atropine 1% once daily to sound eye; plano lens if insufficient response; Patching (6–full waking hours/day); escalate if inadequate response	24	Age 3–<7 years; VA in amblyopic eye 20/40–20/100; ≥3 line interocular difference	NEI (public funding)

Pediatric Eye Disease Investigator Group (2005)	USA	RCT	32 (Near) / 32 (Non-near)	5.0 (Near) / 5.4 (Non-near)	Strabismic, anisometropic, combined	2h patching + near activities vs. 2h patching + non-near activities	4	Amblyopic eye VA 20/40 to 20/400 (logMAR), age 3–<7, ATS protocol, ≥3 logMAR difference	No
Pediatric Eye Disease Investigator Group (2008)	USA (multi-center, 39 sites)	RCT	95 (Atropine) / 98(Patching)	9.0 years overall (not separated)	Moderate amblyopia (20/40–20/100); due to anisometropia and/or strabismus	Atropine 1% (weekend) vs. patching (2h/day) of sound eye	17 weeks primary, follow-up as neede	Age 7.0–<13.0 yrs; VA 20/40–20/100 in amblyopic eye; ≥3-line interocular difference; required optimal spectacle correction for ≥16 weeks prior to randomization	No
Pediatric Eye Disease Investigator Group (2009)	USA (multi-center, 30 sites)	RCT	90 (Atropine +Plano)/ 90 (Atropine Only)	5.1(both groups)	Strabismic, Anisometropic, Combined	Weekend Atropine + Plano lens vs. Weekend Atropine alone	18	Age 3 to <7; Amblyopic eye BCVA 20/40–20/100; Interocular difference ≥3 lines; ≥1.50 D hyperopia in sound eye; ≥16 weeks prior optimal correction or stable VA; sound eye BCVA ≥20/40	No
Pediatric Eye Disease Investigator Group (2010)	USA (PEDIG, multi-site)	RCT	89(Bangerte r) / 97 (Patching)	6.3 (range 3–<10)	Moderate amblyopia due to strabismus (27%), anisometropia (44%), or both (30%)	Bangerter filter vs. daily patching	24	Amblyopic eye VA: 20/40–20/80; fellow eye VA ≥20/40; ≥3-line IOD; current spectacle use, stable VA	No
Pediatric Eye Disease Investigator Group (2013)	USA & UK (PEDIG, multi-center)	RCT	83 (2h) / 86 (6h)	5.9 (both groups)	Residual amblyopia after patching for strabismus (19%), anisometropia (40–49%), or both (31–42%)	2 hours/day patching vs. 6 hours/day patching (after stabilization with 2h patching)	10	Age 3–<8 yrs, VA in amblyopic eye 20/32–20/160 (or 20/32 with ≥3 line IOD), after ≥12 wks of 2h/day patching with stable VA	No
Poltavski et al. (2025)	USA (multi-center, 7 clinics)	RCT	21 (Video Game) / 19 (Patching)	5–18 (mean not specified; analytic cohort only includes completers)	Anisometropic (refractive) only	Barron Vision video game therapy vs. standard eye patching	12	Age 4–18; Amblyopic eye VA ≤20/40 (≥0.30 logMAR); Fellow eye VA ≥20/25 (≤0.10 logMAR); Interocular difference ≥0.3 logMAR (3 lines); ≥16 weeks prior optical correction or stable VA (<0.1 logMAR change in 2 exams ≥4 weeks apart); no amblyopia treatment in prior month; no heterotropia/phoria >10Δ; see article for full details	No
Proudlock et al. (2024)	UK, Greece, Austria, Germany,	RCT	170 (EOT)/ 164 (Early	5.2(EOT)/ 5.4 (Early patching)	Anisometropic, strabismic, mixed	(1) Extended Optical Treatment (EOT): 18 weeks full-time glasses, then	EOT: 18 wks glasses, then up to 24 wks patching	Age 3–8 yrs; newly diagnosed, untreated unilateral amblyopia; interocular BCVA diff ≥0.30 logMAR; refractive error ≥1.5 D SE in	No

	Switzerland (30 centers)		patching)			patching (10 h/day, 6 d/wk, up to 24 weeks)<br>(2) Early patching: 3 weeks glasses, then patching (same regimen)	Early patching: 3 wks glasses, then up to 24 wks patching	≥1 eye or ≥1.0 D anisometropia; no previous amblyopia treatment	
Repka et al. (2007)	USA	RCT	134 (Atropine) / 148 (Patching)	3 to <7	Moderate amblyopia (anisometropic, strabismic, combined)	Atropine 1% daily vs. Patching (≥6h/day)	Up to 104 (2 years)	Age 3–<7 yrs; VA 20/40–20/100; ≤0.5 D myopia in sound eye	NIH/NEI-funded
Roy et al. (2023)	India	RCT	27 (Video game)/ 28 (Patching)	range 5-15 (both groups)	Anisometropic amblyopia	Smartphone-based dichoptic video game (2 h/day) vs. Occlusion (6 h/day)	12	Anisometropic amblyopia, 5–15 years, treatment-naïve, BCVA assessed at baseline	No
Sharif et al. (2019)	Iran	RCT	20 (Fluoxetine) / 15 (Placebo)	21 (Fluox)/ 21 (Placebo)	Anisometropic and mixed amblyopia	Oral fluoxetine + patching vs. placebo + patching	12	Age 10–40 years; anisometropia (hyperopia >1.5D, astigmatism >2D, myopia >3D); BSCVA in amblyopic eye worse than 0.2 logMAR or ≥2-line difference vs. fellow eye	No
Singh et al. (2017)	India	RCT	34 (video game + occlusion) / 34 (occlusion only)	range 6–14 (both groups)	Anisometropic amblyopia	Occlusion (6 h/day) vs. occlusion (6 h/day) + 1 h/day monocular video game play (first month)	12	Age 6–14 y; anisometropic amblyopia; BCVA amblyopic eye >6/36 and <6/12 (approx. 0.78–0.3 logMAR); no manifest strabismus	No
Stanković & Milenković (2007)	Serbia	RCT	26 (Continuous)/ 25 (Alternating)	8.7 (Range: 5.4–26.8)	Unilateral amblyopia due to strabismus or microtropia (with or without anisometropia)	(1) Continuous full-time occlusion of sound eye (24h/day)<br>(2) Alternating full-time occlusion: sound eye (24h/day) for [age+1] days, then amblyopic eye (24h/day) for 1 day	20	Age >5 years; VA in AE ≤0.4 (crowded Landolt Cs); after ≥4 weeks of optimal optical correction; excluded: isolated anisometropic amblyopia, significant myopia, structural/systemic anomalies	No
Stewart et al. (2007)	UK	RCT	40 (6h)/ 40 (12h)	5.4 (6h)/5.6 (12h)	Strabismic, anisometropic, mixed	Occlusion 6 h/d vs. 12 h/d	Up to 26 weeks (mean ~9-10 weeks to best acuity)	Confirmed unilateral amblyopia (≥0.1 logMAR difference); ages 3-8; no prior occlusion; full refractive adaptation required	No
Tejedor & Ogallar (2008)	Spain	RCT	31 (atropine)/ 32 (optical)	5.64 (atropine)/ 6.11 (optical)	Strabismic or anisometropic	(1) Atropine 1% (2x/week)<br>(2) Optical penalization with positive lenses (mean +1.53 D over best correction)	24	Age 2–10 yrs; AE VA ≥0.5 logMAR (≥20/63 Snellen); interocular VA difference ≥2 logMAR lines; no prior amblyopia treatment; no organic pathology	No

Tejedor & Gutiérrez-Carmona (2023)	Spain	RCT	16 (bifocal+atropine)/ 19 (atropine only)	5.6 (bifocal+atropine), 5.6 (atropine)	Hyperopic anisometropic amblyopia	Bifocal lenses + atropine vs. atropine alone	24	Age 4–8 years; hyperopic anisometropia ≥0.75 D; amblyopic eye VA 0.2–0.6 logMAR; sound eye VA 0–0.1 logMAR; untreated, no strabismus or ocular disease	No
Uttamapinan et al. (2024)	Thailand	RCT	22 (App)/ 22 (Control)	7 (App)/ 8(Control)	Strabismic, anisometropic, deprivation, mixed	(A) Occlusion + smartphone app (ATCU)<br>(B) Occlusion + standard care (logbook)	12	Age 4–12 y; unilateral amblyopia (strabismic, anisometropic, deprivation, mixed); BCVA 20/40–20/400; ≥2-line difference; prior spectacle/occlusion allowed	No
Wang et al. (2016)	USA	RCT	19 (IO) / 15 (Patching)	5.7 (IO) / 5.9 (Patch)	Moderate unilateral (strabismic, anisometropic or mixed)	Intermittent Occlusion Glasses (Amblyz) vs. Patching	12	VA in amblyopic eye 20/40 to 20/100, interocular difference ≥2 lines, ≥12 weeks prior optical correction	Amblyz provided by XPAND 3D Group; no role in design or analysis
Wang et al. (2021)	China	RCT	53 (CAPT) / 55 (patching only)	5.2 (CAPT); 5.1 (patching)	Strabismic, anisometropic, or mixed	Combined atropine (1% eye gel) and patching (6 h/day) vs. patching alone (6 h/day); both groups with near activitie	24	Age 3–12 y; severe amblyopia (20/100 to 20/500, ≥2 line interocular difference); no prior amblyopia treatment except ≤1 month refractive correction; strabismic, anisometropic, or mixed amblyopia	No
Wu et al. (2010)	Canada	RCT	8 (patching + telescope) / 7 (patching only)	7.1(patch+tel) / 8.7 (patch only)	Strabismic, anisometropic, mixed mechanism, high-isometropic hyperopic	Patching + telescope vs. patching only (both 30 min/day fellow eye)	17	Age 4–17 y; strabismic, anisometropic, mixed, or high-isometropic hyperopic amblyopia; VA (amblyopic eye) 0.3–1.3 logMAR (20/40–20/400); ≥0.3 logMAR interocular difference; failed previous tx	No
Wygnanski-Jaffe et al. (2023)	Israel (6 centers)	RCT	51 (CureSight) 52 (Patching)	6.63 (CureSight) 6.94 (Patching)	Anisometropic, small-angle strabismic, or mixed amblyopia	(1) CureSight binocular treatment (eye-tracking, home-based, 90 min/day, 5 days/wk, 16 wks, 120h total)<br>(2) Patching (2 h/day, 7 days/wk, 16 wks, 224h total)	16	Age 4–<9 yrs; BCVA (amblyopic eye) 20/32–20/100; fellow eye BCVA ≥20/40 (age 4–5) or ≥20/32 (age ≥5); interocular diff ≥2 lines; stable VA after refractive adaptation; anisometropia ≥1.0 D SE or ≥1.5 D astigmatism; strabismus ≤5 PD	No
Wygnanski-Jaffe et al. (2025)	Israel, USA	RCT	75 (dichoptic) / 74 (patching)	~6.4 years (range 4–9, similar in both groups)	Anisometropic, small-angle strabismic, mixed	Binocular dichoptic (CureSight: 90 min/day, 5 days/week) vs. patching (2 h/day, 7 days/week)	16	Interocular difference ≥2 logMAR lines; age 4–<9 years; baseline VA ~0.4 logMAR	No
Yuan et al. (2021)	China	RCT	20 (AFG) / 20 (Patching)	10.0 (AFG) / 9.05 (Patching)	Anisometropic	Alternative Flicker Glass (1h/day) vs. Patching (2h/day)	12	BCVA 0.70–0.20 logMAR in amblyopic eye; ≥2 lines interocular difference; no treatment in previous 3 months; refractive adaptation for 12 weeks	No

Zhao et al. (2010)	China, Hong Kong, India, USA	RCT	43 (Acupuncture) / 45 (Patching)	9.4 (Acupuncture) / 9.4 (Patching)	Anisometropic	Acupuncture vs. 2h/day patching (both + optical correction and near activity)	15 (primary outcome), 25 (follow-up)	Age 7–12 y, anisometropia ≥0.5 D SE or ≥1.5 D astigmatism, BSCVA 0.3–0.8 logMAR in amblyopic eye, interocular VA difference ≥2 lines, no prior amblyopia tx except specs	Several authors filed provisional patent for acupuncture method.
Zhu et al. (2023)	China (Dalian)	RCT	18 (Combined group)/ 14 (Patching)	6.90(Combined group)/6.65 (Patching)	Anisometropic (75%), Strabismic (22%), Combined (3%)	(1) Part-time patching + binocular therapy (3D movies + Bangerter blur)<br>(2) Part-time patching only	6	Age 5–12; BCVA in amblyopic eye worse than 0.2 logMAR, fellow eye ≤0.1 logMAR, interocular difference ≥0.2 logMAR; >16 weeks prior refractive correction; residual deficit after patching	No

ADP (Alternate-Day Patching), AE (Amblyopic Eye), AFG (Alternative Flicker Glass), AMTP (Asynchronous Movie Training Program), App (Application), ATCU (Amblyopia Treatment Compliance Utility), BCVA (Best-Corrected Visual Acuity), BBV (Balanced Binocular Viewing), BSCVA (Best Spectacle-Corrected Visual Acuity), CAM (CAM Vision Stimulator), CAPT (Combined Atropine and Patching Therapy), CDP-choline (Cytidine Diphosphate Choline), CDVA (Corrected Distance Visual Acuity), CMAC (Chinese Medical Association Criteria), COI (Conflict of Interest), Control (Control Group), DVA (Distance Visual Acuity), EFG (Eyetronix Flicker Glass), EOT (Extended Optical Treatment), h/day (hours per day), ICL (Implantable Collamer Lens), IOD (Interocular Difference), IO (Intermittent Occlusion Glasses), IPS (Intermittent Photic Stimulation), logMAR (Logarithm of the Minimum Angle of Resolution), NIH (National Institutes of Health), NEI (National Eye Institute), OCL (Occluding Contact Lens), PEDIG (Pediatric Eye Disease Investigator Group), PD (Prism Diopters), PL (Perceptual Learning), RCT (Randomized Controlled Trial), rTMS (repetitive Transcranial Magnetic Stimulation), SE (Spherical Equivalent), VA (Visual Acuity), VR (Virtual Reality), wks (weeks).
